# Direct and Auger Electron-Induced, Single- and Double-Strand Breaks on Plasmid DNA Caused by ^99m^Tc-Labeled Pyrene Derivatives and the Effect of Bonding Distance

**DOI:** 10.1371/journal.pone.0161973

**Published:** 2016-09-01

**Authors:** Falco Reissig, Constantin Mamat, Joerg Steinbach, Hans-Juergen Pietzsch, Robert Freudenberg, Carlos Navarro-Retamal, Julio Caballero, Joerg Kotzerke, Gerd Wunderlich

**Affiliations:** 1 University Hospital/ Faculty of Medicine Carl Gustav Carus, Technische Universität Dresden, Department of Nuclear Medicine, Dresden, Germany; 2 Helmholtz-Zentrum Dresden-Rossendorf, Institute of Radiopharmaceutical Cancer Research, Dresden, Germany; 3 Centro de Bioinformática y Simulación Molecular, Facultad de Ingeniería, Universidad de Talca, 2 Norte 685, Casilla 721, Talca, Chile; National Center for Toxicological Research, UNITED STATES

## Abstract

It is evident that ^99m^Tc causes radical-mediated DNA damage due to Auger electrons, which were emitted simultaneously with the known γ-emission of ^99m^Tc. We have synthesized a series of new ^99m^Tc-labeled pyrene derivatives with varied distances between the pyrene moiety and the radionuclide. The pyrene motif is a common DNA intercalator and allowed us to test the influence of the radionuclide distance on damages of the DNA helix. In general, pUC 19 plasmid DNA enables the investigation of the unprotected interactions between the radiotracers and DNA that results in single-strand breaks (SSB) or double-strand breaks (DSB). The resulting DNA fragments were separated by gel electrophoresis and quantified by fluorescent staining. Direct DNA damage and radical-induced indirect DNA damage by radiolysis products of water were evaluated in the presence or absence of the radical scavenger DMSO. We demonstrated that Auger electrons directly induced both SSB and DSB in high efficiency when ^99m^Tc was tightly bound to the plasmid DNA and this damage could not be completely prevented by DMSO, a free radical scavenger. For the first time, we were able to minimize this effect by increasing the carbon chain lengths between the pyrene moiety and the ^99m^Tc nuclide. However, a critical distance between the ^99m^Tc atom and the DNA helix could not be determined due to the significantly lowered DSB generation resulting from the interaction which is dependent on the type of the ^99m^Tc binding motif. The effect of variable DNA damage caused by the different chain length between the pyrene residue and the Tc-core as well as the possible conformations of the applied Tc-complexes was supplemented with molecular dynamics (MD) calculations. The effectiveness of the DNA-binding ^99m^Tc-labeled pyrene derivatives was demonstrated by comparison to non-DNA-binding ^99m^TcO_4_^–^, since nearly all DNA damage caused by ^99m^TcO_4_^–^ was prevented by incubating with DMSO.

## Introduction

^99m^Tc is commonly used as a γ-emitter for imaging purposes in nuclear medicine. Due to its decay properties (γ-radiation at 141 keV, t_1/2_ = 6.0 h) it can be detected using SPECT (single photon emission computer tomography) imaging techniques. In addition to the imageable 141 keV γ-emission, ^99m^Tc also converts by internal conversion resulting in emission of low energy Auger and conversion electrons (see Table A in S1 File). However, the resulting effects due to these Auger electron emissions are still controversial [1, 2].

More than 50% of all radionuclides decay by internal conversion or/and electron capture causing the emission of a cascade of very low energy electrons. This phenomenon is known as the Auger effect. The main advantage of Auger electrons is that they behave like high LET (linear energy transfer) radiation despite their low emission energy. Therefore, Auger electrons can induce various types of DNA damage such as multiple double-strand breaks (DSB) when they are localized in close proximity to the DNA [3–6]. The diagnostic and therapeutic use of Auger emitters, especially ^125^I (^123^I) or ^111^In, has been widely discussed [7–10]. In addition to these prominent Auger emitters, ^99m^Tc emits almost five low energy electrons with a range of less than 200 nm [1, 11] and initial tests demonstrated that it might be possible to use ^99m^Tc for Auger electron therapy [12, 13]. Cell experiments proved that Auger electrons emitted by ^99m^Tc can initiate cell death [14] but the damage was not clearly quantified.

The use of double-stranded plasmid DNA as a biological model enables the investigation of unprotected interactions between molecules and supercoiled DNA (SC) allowing the distinction between single-strand breaks (SSB) and double-strand breaks. The appearance of open circle DNA (OC) is commonly used as an indicator of SSB and linear DNA (L) is the result of DSB.

Previous studies on the therapeutic effect of Auger electrons from ^99m^Tc provided information about the DNA damage caused by ^99m^Tc interaction with plasmid DNA [15][16].To better understand the biophysical mechanisms underlying the induction of DNA single- and double-strand breaks caused by the emitted Auger electrons, we translated Balagurumoorthy’s studies regarding to the effect of distance between ^125^I-labeled Hoechst derivatives and plasmid DNA to ^99m^Tc. They were able to demonstrate the damage of DNA provoked by Auger electron emitters especially of ^125^I in dependance on the distance between the radionuclide and the DNA strand [3, 17].

In most cases, radioiodine was covalently bound to several intercalating molecules [3, 18]. Conversely, ^99m^Tc is exclusively bound to the molecules via complexation using chelating compounds; a direct covalent bond to the Tc is not feasible. Therefore, a tridentate chelating unit to bind the ^99m^Tc- (or ^186,188^Re)-tricarbonyl moiety has become a well established system for the complexation of Tc and Re radionuclides [19–21]. Additionally, pyrene is known to be a DNA intercalating molecule [12]. For these reasons, we designed and synthesized four new pyrene derivatives with different-sized linkers using a tridentate chelating unit for complexation of ^99m^Tc.

The primary aim of our study was to learn more about the properties of Auger electrons emitted by ^99m^Tc. To achieve this aim, we prepared a ^99m^Tc-complex with an optimal distance between the ^99m^Tc(CO)_3_-core and the pyrene residue to position the ^99m^Tc in close proximity to the plasmid DNA to induce direct SSB and DSB. By increasing the distance between the DNA-intercalating moiety and the bonding moiety for ^99m^Tc, we expected to observe decrease of direct DNA damages. This distance dependance has not been reported for ^99m^Tc until now. Additionally, we sought to differentiate between indirect effects of radicals caused by the radiolysis of water and direct destruction caused by Auger electrons to DNA. Therefore, DMSO, a commonly known radical scavenger, was incubated in a second set of experiments to illustrate the effect on DNA damage [22]. Finally, we compared the effects of the DNA-binding ^99m^Tc pyrene derivatives with that of non-DNA binding ^99m^TcO_4_^–^.

## Materials and Methods

Pyrene **9** was synthesized according to Hafliger et al. [12], compound **6a** was prepared according to Pretze and Mamat [23] and **6b** was prepared according to Adres-Guisot et al. [24]. All syntheses of the pyrene chelators as well as the radiolabeling procedures can be found in detail in the Supporting Information (S1 File).

### pUC 19 plasmid DNA

The pUC 19 plasmid (2686 base pairs) was purchased from New England Biolabs (Ipswich, UK). The DNA stock solution was diluted in TE-Buffer (10 mM TRIS-HCl, 1 mM EDTA, pH 7.5) to obtain a final concentration of 0.1 μg/μL (aliquots were stored at -20°C). Only samples containing more than 95% of supercoiled plasmid DNA were used for our studies. To obtain linear plasmid DNA, pUC 19 was incubated with BamHI (Invitrogen, Karlsruhe, Germany).

### Incubation procedure and gel electrophoresis

The total volume of each incubation was brought up to 20 μL with purified water. All samples contained 200 ng of pUC 19 plasmid DNA. The samples were incubated in 1.5 mL microtubes (Sarstedt, Nümbrecht, Germany). Samples only containing either supercoiled or linear pUC 19 were used as controls for the plasmid DNA.

The respective complexes [^99m^Tc]**Ia**, [^99m^Tc]**Ib**, [^99m^Tc]**II**, [^99m^Tc]**III** with varied ^99m^Tc activites (3–15 MBq in steps of 3 MBq; 1 GBq/mL in 0.9% NaCl solution) were added to the plasmid solutions. For comparison, ^99m^TcO_4_^–^ was added as a reference to the DNA solutions (3–15 MBq in steps of 3 MBq; 1 GBq/mL; corresponding to a dose of approximately 10–50 Gy after 24 hours). Each sample was incubated for 24 h at room temperature.

For characterizing the DNA damage, a second set of experiments was performed as described above except the samples were incubated with DMSO (Roth, Karlsruhe, Germany) instead of water with a final concentration of 0.2 M DMSO.

After irradiation, 1.25 μL of blue juice gel loading buffer (Invitrogen, Karlsruhe, Germany) was added to 10 μL of each sample. The samples were then pipetted in wells of a 1.4% agarose-gel in Tris-Acetate-EDTA buffer (TAE buffer, Sigma Aldrich). Gel electrophoresis (Sub Cell GT, BioRad, Munich, Germany) was run at 120 V for 100 min. After electrophoresis, the gel was stained with an ethidium bromide solution (0.5 μg/mL) for 30 min and three different conformations of pUC 19 plasmid DNA were identifiable (SC, OC and L). Each fraction resulted in different DNA bands and were separated by electrophoresis due to their different sizes and loadings.

The different conformation forms of pUC 19 were visualized by using an UV transilluminator (Diana III Digital Imaging System, Raytest, Straubenhardt, Germany). The DNA bands were imaged by a charge-coupled device (CCD) camera. For the interpretation of our results, we assumed that the sum of SC, OC and L plasmid fraction equaled 100%. The SC plasmid band was the native one. If there was any DNA damage, two other conformations appeared. The OC plasmid DNA corresponds to the fraction of induced SSB, while the L plasmid fraction corresponds to the fraction of induced DSB. The DNA damage was quantified by integrating the fluorescing intensities of the three possible DNA conformations. The yields of each conformation form of pUC 19 were quantified by integrating the corresponding fluorescing intensities by using the open-source platform software Fiji [25].

### Calculations of SSB and DSB

The SSB and DSB calculations were based on the assumption that the binding of ^99m^Tc-labeled pyrene derivatives to DNA and thus the strand breaks follow a Poisson distribution. The average of SSB (X_SSB_) and DSB (X_DSB_) was calculated from the fraction of linear DNA (F_L_) and the remaining supercoiled DNA fraction (F_SC_) determined for each sample with a defined amount of activity.

$${\text{X}}_{\text{SSB}}=\text{ln}\left[\left(1-{\text{F}}_{\text{L}}\right)/{\text{F}}_{\text{SC}}\right]$$ (1)

$${\text{X}}_{\text{DSB}}={\text{F}}_{\text{L}}/\left(1-{\text{F}}_{\text{L}}\right)$$ (2)

Assuming that 200 ng of pUC 19 plasmid DNA contain 6.88·10^10^ plasmid molecules, the calculation of SSB and DSB per plasmid are as follows:

$${\text{X}}_{\text{SSB}/\text{plasmid}}=\frac{{\text{X}}_{\text{SSB}}}{6.88\cdot 10^{10}}$$ (3)

$${\text{X}}_{\text{DSB}/\text{plasmid}}=\frac{{\text{X}}_{\text{DSB}}}{6.88\cdot 10^{10}}$$ (4)

Incubating an activity of 15 MBq of ^99m^Tc-labeled pyrene derivatives or ^99m^TcO_4_^–^, respectively, within an incubation time of 24 h resulted in a total amount of 4.38·10^11^ decays. Refering the total number of decays within 24 h to the total number of plasmid DNA molecules leads to an amount of 6.37 decays per plasmid molecule. Combining all information, it is possible to calculate the yield of SSB (Y_SSB_) and DSB (Y_DSB_) per plasmid molecule per decay as follows.

$${\text{Y}}_{\text{SSB}}=\frac{{\text{X}}_{\text{SSB}/\text{plasmid}}}{6.37}$$ (5)

$${\text{Y}}_{\text{DSB}}=\frac{{\text{X}}_{\text{DSB}/\text{plasmid}}}{6.37}$$ (6)

## Results

### Preparation of the chelators

**Fig 1:**
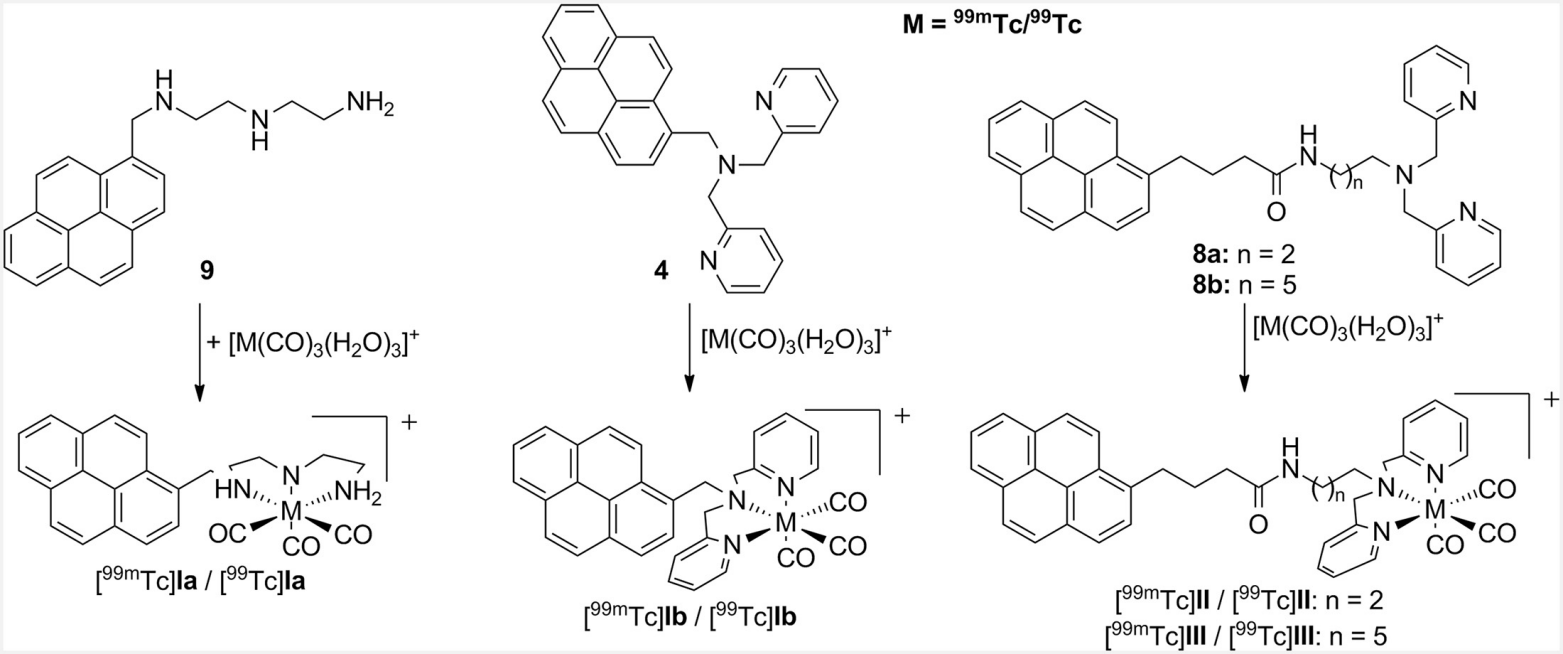
Overview of the synthesized unlabeled compounds, radiolabeling procedures and the nomenclature of the ^99m^Tc-radiolabeled pyrene derivatives (M = ^99m^Tc/^99^Tc).

To study the dependance on the distance of the Tc(CO)_3_-core to the DNA, three pyrene derivatives were chosen and compared regarding the influence of DNA damage and association to different chain lengths between the pyrene moiety and the radionuclide. In this case, the dipicolylamine (DPA) residue acts as tridentate ligand, which is known to be an excellent chelator for the Tc(CO)_3_ as well as for the Re(CO)_3_ core [26]. The first pyrene derivative **4** was prepared according to a modified synthesis procedure [27] from compound **3**, which was reacted with 2-(chloromethyl)pyridine to yield the desired pyrene derivative **4** in 70% yield. The preparation of the pyrene derivatives **8a** and **8b** with longer alkyl chains was realized using two azides **7a,b** which were obtained from the reaction of **5** with azidoalkyl tosylates **6a** and **6b**, respectively, in yields of 59% and 55%. In the next step, compounds **7a,b** were first treated with triphenyl phosphane at 60°C for 3 h. Subsequently, 1-pyrenebutyric acid *N*-hydroxysuccinimide ester was added at ambient temperature to this mixture and the desired pyrenes **8a,b** were obtained in yields of 57% and 61%, respectively. The reaction path is pointed out in Fig 1. Further details can be found in the Supporting Information (S1 File).

In addition to the three pyrene derivatives **4**, **8a** and **8b**, a literature known pyrene **9** with an aliphatic tridentate binding moiety for the Tc(CO)_3_-core was synthesized [12].

### Radiolabeling, analysis and purification of the ^99m^Tc-labeled pyrene derivatives

For a clearer view concerning the biological experiments, the radiolabeled substances were named as pyrenes [^99m^Tc]**Ia** / [^99m^Tc]**Ib** / [^99m^Tc]**II** / [^99m^Tc]**III** (Fig 1).

**Fig 2:**
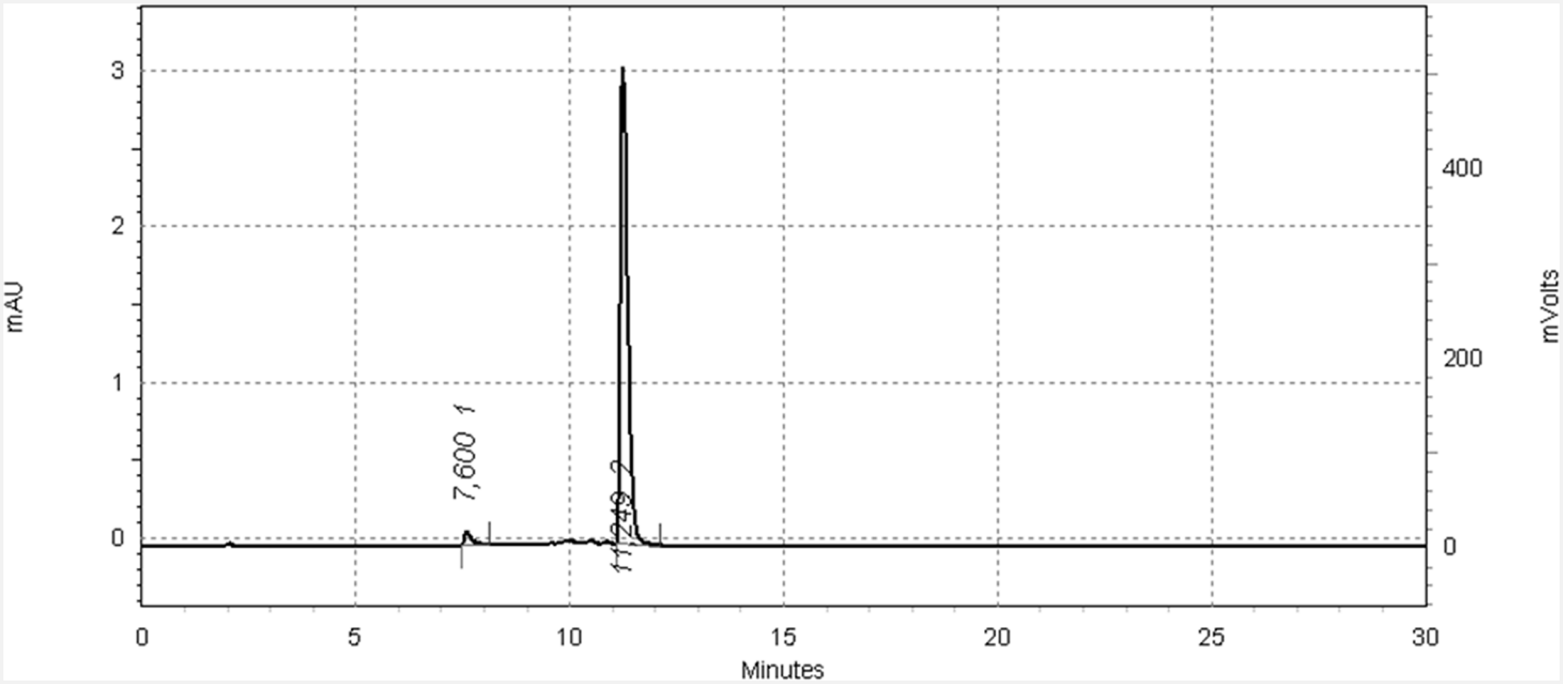
Representative radio-HPLC chromatogram of [^99m^Tc]Ib after radiolabeling and purification by HPLC; radioactive peak is shown at *t*_R_ = 11.3 min with RCP = 97% (HPLC-gradient: 5/95% A/B to 95/5% A/B within 11 min; A: H_2_O (0.05% TFA) B: acetonitrile (0.05% TFA).

All radiolabeled derivatives were analyzed and purified by HPLC. It was possible to separate the radiolabeled product from the initial pyrene derivative to ensure a maximum specific activity of the product. The radiochemical purity of the isolated final products was greater than 95%, a representative chromatogram is shown in Fig 2. A summary of the HPLC retention times of the chelators **4**, **8a**, **8b** and **9** in comparison with the radiolabeled complexes [^99m^Tc]**Ia**, [^99m^Tc]**Ib**, [^99m^Tc]**II**, [^99m^Tc]**III** is shown in Table 1.

**Table 1:** Summary of HPLC retention times (*t*_R_) of chelators 4, 8a, 8b, 9 and the radiolabeled pyrene derivatives [^99m^Tc]Ia, [^99m^Tc]Ib, [^99m^Tc]II, [^99m^Tc]III in comparison with [^99m^Tc(CO)_3_(H_2_O)_3_]^+^ and ^99m^TcO4– as references.

Chelator	*t*_R_ [min] (UV-trace)	Complex	*t*_R_ [min] (γ-trace)
**9**	7.0	[^99m^Tc]**Ia**	9.9, 10.2*
**4**	8.5	[^99m^Tc]**Ib**	11.3
**8a**	9.1	[^99m^Tc]**II**	10.8
**8b**	9.8	[^99m^Tc]**III**	11.3
		[^99m^Tc(CO)_3_(H_2_O)_3_]^+^	2.3
		^99m^TcO_4_^–^	2.0

* = two isomers were obtained

### Quantification of plasmid DNA damage

**Fig 3:**
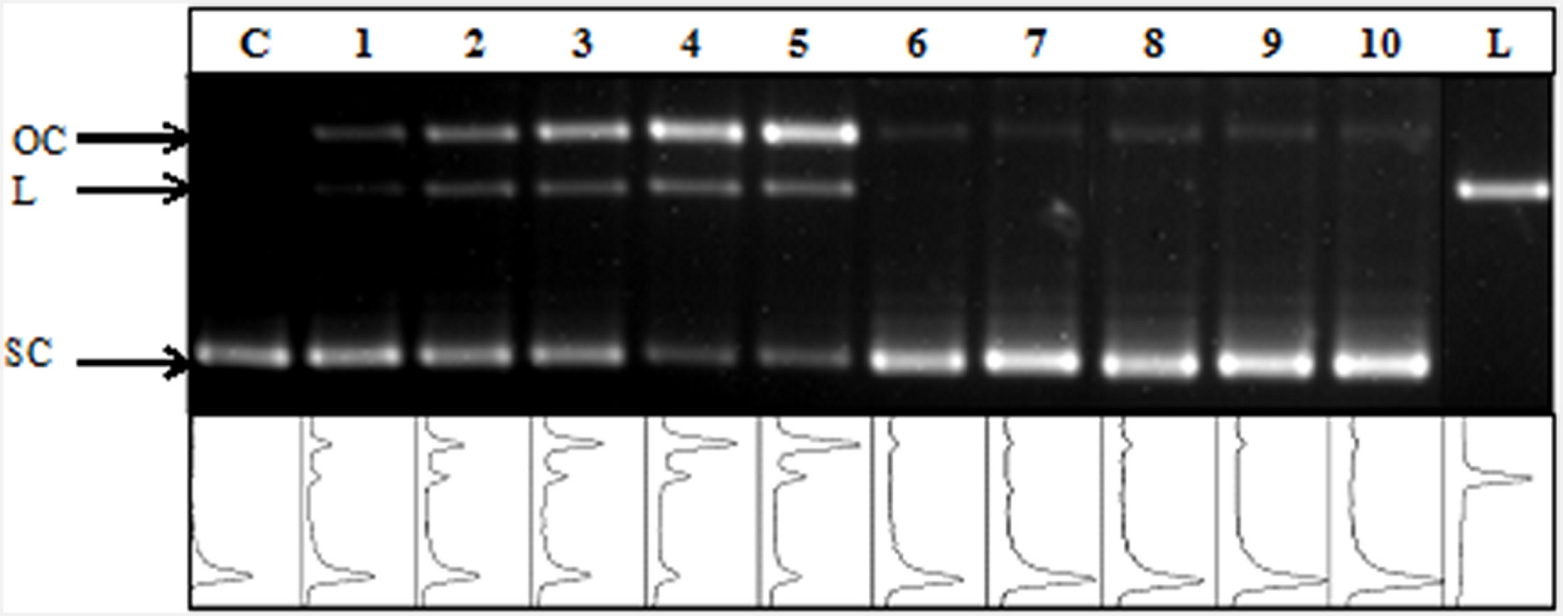
Representative agarose gel of supercoiled (SC) pUC 19 plasmid DNA incubated with various activities of [^99m^Tc]Ib and [^99m^Tc]III in presence of DMSO (0.2 M). Lane C is the control pUC 19 plasmid DNA (SC) and lane L is the linear control pUC 19 plasmid DNA (L). OC represent open circular conformation of pUC 19 plasmid DNA. Lanes 1–5 show pUC 19 plasmid DNA incubated with [^99m^Tc]Ib (3–15 MBq). Lanes 6–10 show pUC 19 plasmid DNA incubated with [^99m^Tc]III (3–15 MBq).

The binding of [^99m^Tc]**Ia**, [^99m^Tc]**Ib**, [^99m^Tc]**II**, [^99m^Tc]**III** was shown by quantifiying the direct DNA damage of pUC 19 plasmid DNA as either SSB or DSB. In principle, pUC 19 was able to convert to three different conformations (OC, L, SC). A representative agarose gel (Fig 3) shows the incubation of two different ^99m^Tc-pyrene derivatives [^99m^Tc]**Ib** and [^99m^Tc]**III** in the presence of 0.2 M DMSO. Lanes that are incubated with derivative [^99m^Tc]**Ib** (1–5) showed more induced OC (SSB) and L (DSB) in contrast to lanes which are incubated with [^99m^Tc]**III** in lanes 6–10. The strong differences were visible even without any quantification of fluorescence intensities.

Concerning induced SSB and DSB, the smaller pyrene derivatives ([^99m^Tc]**Ia** and [^99m^Tc]**Ib**) were more effective than the pyrene derivatives with longer alkyl chains ([^99m^Tc]**II** and [^99m^Tc]**III**) (Table 2). However, the type of chelators used in [^99m^Tc]**Ia** and [^99m^Tc]**Ib** had an influence on the number of observed SSB and DSB. Moreover, the alkyl chain length in [^99m^Tc]**II** and [^99m^Tc]**III** had no further influence. Tables 2 and 3 give an overview of the detected OC and L that represents SSB and DSB per plasmid molecule and decay for an incubated activity of 15 MBq of [^99m^Tc]**Ia**, [^99m^Tc]**Ib**, [^99m^Tc]**II**, [^99m^Tc]**III**, and ^99m^TcO_4_^–^ in absence (–) and presence (+) of 0.2 M DMSO, respectively. All values are reported as the mean value of three independent trials with the experimental standard deviation given.

**Table 2:** Summary of quantitative analysis of the fractions of OC (SSB) and L (DSB) caused by incubation of 15 MBq [^99m^Tc]Ia, [^99m^Tc]Ib, [^99m^Tc]II, [^99m^Tc]III, and ^99m^TcO4– in absence (–) and presence (+) of 0.2 M DMSO.

	(–) DMSO		(+) 0.2 M DMSO	
Compound	OC [%]	L [%]	OC [%]	L [%]
^99m^TcO_4_^–^	89 ± 1	10 ± 1	9 ± 1	<1
[^99m^Tc]**Ia**	84 ± 3	16 ± 3	37 ± 8	12 ± 5
[^99m^Tc**]Ib**	64 ± 5	23 ± 4	61 ± 2	22 ± 7
[^99m^Tc]**II**	7 ± 4	<1	8 ± 2	<1
[^99m^Tc]**III**	5 ± 1	<1	4 ± 1	<1

**Table 3:** Calculation of the average yields of SSB and DSB per plasmid molecule and per decay (Y_SSB_ and Y_DSB_) of ^99m^Tc by incubation of 15 MBq [^99m^Tc]Ia, [^99m^Tc]Ib, [^99m^Tc]II, [^99m^Tc]III, and ^99m^TcO4– in absence (–) and presence (+) of 0.2 M DMSO.

	(–) DMSO		(+) 0.2 M DMSO	
Compound	Y_SSB_	Y_DSB_	Y_SSB_	Y_DSB_
^99m^TcO_4_^–^	0.68 ± 0.20	0.017 ± 0.0006	0.015 ± 0.001	<0.0016
[^99m^Tc]**Ia**	—*	0.030 ± 0.007	0.087 ± 0.050	0.022 ± 0.011
[^99m^Tc]**Ib**	0.28 ± 0.11	0.047 ± 0.010	0.241 ± 0.092	0.044 ± 0.017
[^99m^Tc]**II**	0.011 ± 0.006	<0.0016	0.012 ± 0.004	<0.0016
[^99m^Tc]**III**	0.009 ± 0.0001	<0.0016	0.007 ± 0.0001	<0.0016

* sc fraction was non-detectable.

On the basis of the data of Tables 2 and 3, we calculated the yields of SSB and DSB per decay. By incubating 0.2 M DMSO, we ensure that the direct damage results only from the Auger electrons by minimizing the DNA damage caused by radical formation. We calculated a direct SSB yield of 0.09 per plasmid and decay as well as a DSB yield of 0.02 per plasmid and decay for [^99m^Tc]**Ia**. Further, a SSB yield of 0.24 per plasmid and decay as well as a DSB yield of 0.04 per plasmid and decay was calculated for [^99m^Tc]**Ib**. For [^99m^Tc]**II**, [^99m^Tc]**III** and ^99m^TcO_4_^–^, there was no L plasmid fraction detectable. Thus, no DSB was found and the SSB yield of these experiments was about 0.01 per plasmid and decay.

### Decrease of native SC plasmid fraction

**Fig 4:**
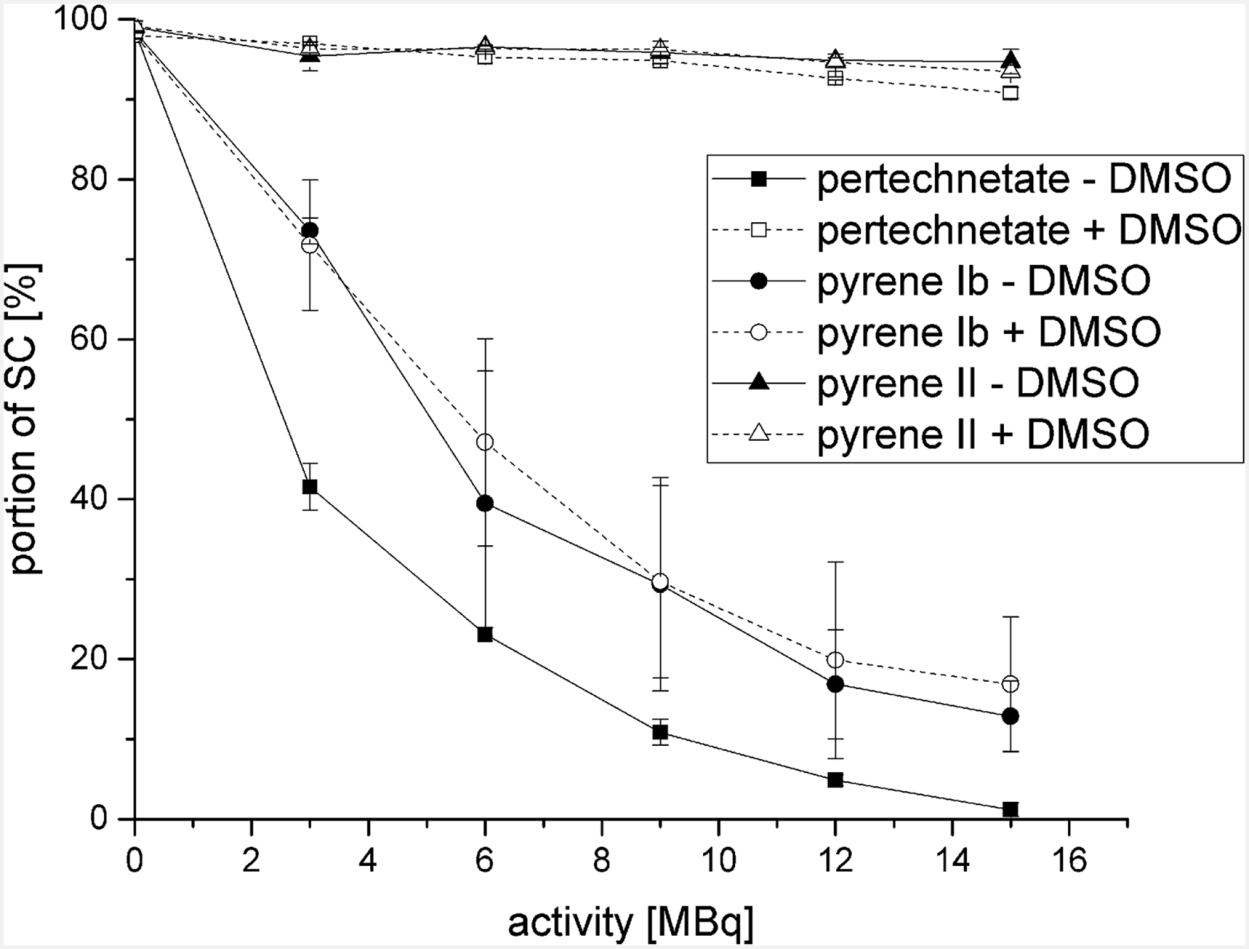
Quantitative analysis of agarose gel electrophoresis for determination of decreasing SC plasmid fraction in absence (–) and presence (+) of 0.2 M DMSO. Incubation of pUC 19 plasmid DNA with 3–15 MBq of [^99m^Tc]Ib and [^99m^Tc]II, and ^99m^TcO_4_^–^ for 24 h at room temperature.

The results in Fig 4 represent quantified data regarding to the decrease of intact plasmid DNA (SC) in relation to the incubated activity. A protective effect of the DMSO against the damages caused from ^99m^TcO_4_^–^ was obvious. However, this was not observed in combination with [^99m^Tc]**Ib** and [^99m^Tc]**II**. It was likely that these pyrene derivatives acted similar to DMSO as free radical scavengers. For this reason further experiments were performed to prove this.

### Pyrene derivatives working as free radical scavengers

To test the capacity of the pyrene derivatives to scavenge free radicals, we incubated plasmid DNA with the lowest (3 MBq) and the highest (15 MBq) activities comparing ^99m^TcO_4_^–^, ^99m^TcO_4_^–^ with DMSO, and ^99m^TcO_4_^–^ with an equivalent amount of each ^99^Tc-labeled pyrene derivative. It is shown in Table 4 that all pyrene derivatives functioned as radical scavengers similar to DMSO. All values are reported as the mean value of three independent trials with the experimental standard deviation given.

**Table 4:** Overview of the detected OC (SSB) and L (DSB) plasmid fractions regarding to the radical scavenging qualities of the ^99^Tc-labeled pyrene derivatives [^99^Tc]Ia, [^99^Tc]Ib, [^99^Tc]II, and [^99^Tc]III in comparison to 0.2 M DMSO. Incubation of pUC 19 plasmid DNA with 3 MBq ^99m^TcO_4_^–^ and 3 MBq equivalent amount of the respective ^99^Tc-labeled pyrene derivative as well as 15 MBq ^99m^TcO_4_^–^ and 15 MBq equivalent amount of the respective ^99^Tc-labeled pyrene derivative.

	3 MBq		15 MBq	
Compound	OC [%]	L [%]	OC [%]	L [%]
^99m^TcO_4_^–^	57 ± 3	3 ± 1	89 ± 1	10 ± 1
^99m^TcO_4_^–^ + 0.2 M DMSO	3 ± 1	<1	9 ± 1	<1
^99m^TcO_4_^–^ + [^99^Tc]**Ia**	6 ± 2	<1	9 ± 1	<1
^99m^TcO_4_^–^ + [^99^Tc]**Ib**	5 ± 3	<1	6 ± 1	<1
^99m^TcO_4_^–^ + [^99^Tc]**II**	8 ± 3	<1	7 ± 2	<1
^99m^TcO_4_^–^ + [^99^Tc]**III**	6 ± 2	<1	10 ± 2	<1

It was conclusively shown in Fig 4, that DMSO has no further influence on the DNA damage during the incubation of pyrene derivative [^99m^Tc]**II**. Relating to the effect of pyrene [^99m^Tc]**Ib**, it was proven that the DNA damage of pyrene [^99m^Tc]**Ib** was not induced indirectly, but directly.

### Increase of open circular and linear plasmid conformation

**Fig 5:**
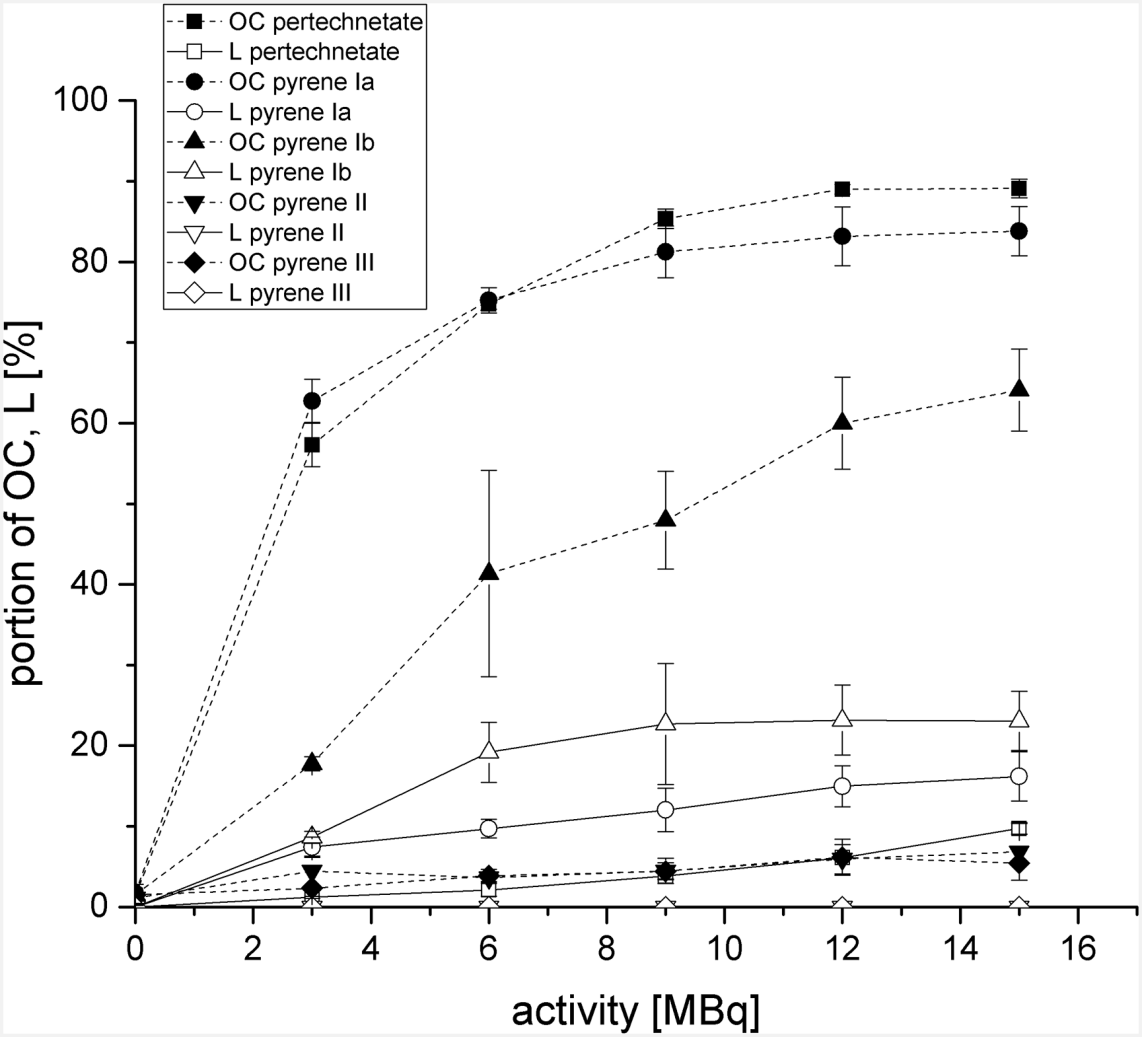
Quantitative analysis of agarose gel electrophoresis for determination of single-strand breaks and double-strand breaks by plotting open circular (OC) and linear (L) plasmid fractions in absence of 0.2 M DMSO. Incubation of pUC 19 plasmid DNA with 3–15 MBq of [^99m^Tc]Ia, [^99m^Tc]Ib, [^99m^Tc]II, [^99m^Tc]III, and ^99m^TcO_4_^–^ reference for 24 h at room temperature.

The increase of induced SSB and DSB as a function of added activity is illustrated in Fig 5 as noted by the increased fractions of OC and L of the pUC 19 plasmid DNA with increased activities. It was observed that ^99m^TcO_4_^–^ induced the highest quantity of SSB and even a small percentage of DSB (≤ 10%). It was also shown that pyrene [^99m^Tc]**Ib** induces a high number of DSB (L). Concerning the other pyrene derivatives there was no higher DNA damage (L) in relation to the incubation of ^99m^TcO_4_^–^ detectable. Due to the radical scavenging effects of the pyrene derivatives themselves which were shown in Table 4, we found that pyrene [^99m^Tc]**Ib** was the only pyrene derivative facilitating a higher number of induced DNA damage (L) regarding induced DSB in contrast to ^99m^TcO_4_^–^.

**Fig 6:**
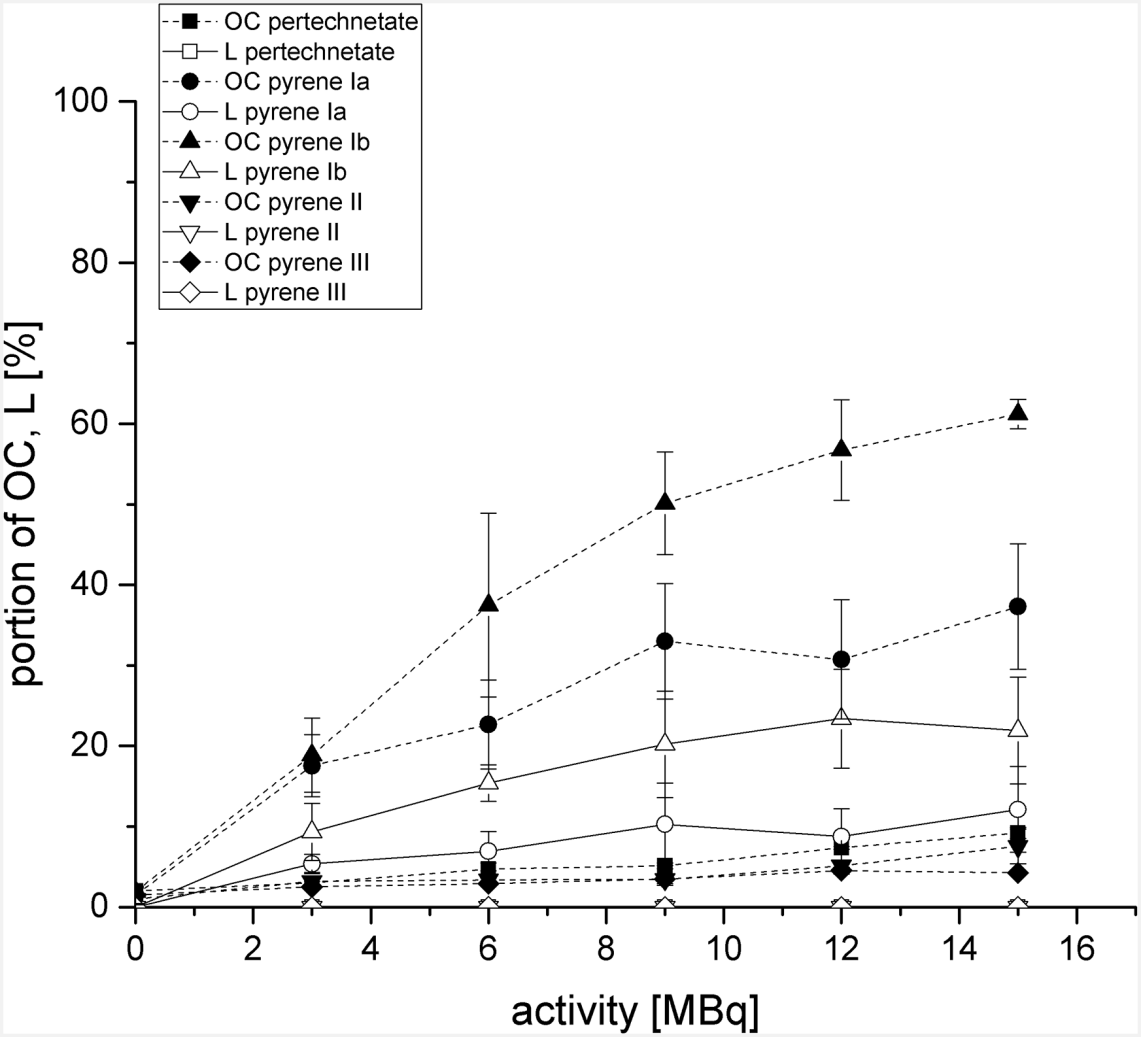
Quantitative analysis of agarose gel electrophoresis for quantification of single-strand breaks and double-strand brekas by plotting open circular (OC) and linear (L) plasmid fractions in presence of 0.2 M DMSO. PUC 19 plasmid DNA was incubated with [^99m^Tc]Ia, [^99m^Tc]Ib, [^99m^Tc]II, [^99m^Tc]III, and 3–15 MBq ^99m^TcO_4_^–^ as reference.

To identify whether the induced SSB and DSB by the [^99m^Tc]pyrenes were caused directly or indirectly (e.g. by radiolysis of water) we performed the same experiments and incubated 0.2 M DMSO additionally as a free radical scavenger. All resulting data is illustrated in Fig 6.

The results in Fig 6 clearly indicate that in case of ^99m^TcO_4_^–^ the additional incubation of 0.2 M DMSO led to a total reduction of DSB and the bulk of SSB as expected. Moreover, 0.2 M DMSO had no influence on the results of ^99m^Tc-labeled pyrene derivatives [^99m^Tc]**Ib**, [^99m^Tc]**II**, and [^99m^Tc]**III**. The plasmid DNA damage (OC and L) was at a constant high level when incubated with pyrene [^99m^Tc]**Ib**, and at a constant low level when incubated with pyrenes [^99m^Tc]**II** and [^99m^Tc]**III** in comparison to incubations without DMSO.

## Discussion

In general, pyrenes act as DNA intercalators, but the unlabeled pyrene derivatives **4**, **8a**, **8b**, and **9** are non-toxic at the low concentrations used in our experiments (data not shown).

The results of incubating pUC 19 plasmid DNA with four different ^99m^Tc-labeled pyrene derivatives [^99m^Tc]**Ia**, [^99m^Tc]**Ib**, [^99m^Tc]**II**, [^99m^Tc]**III** showed a decreasing DNA damage with enhanced distance from about 0.3 nm to 1.5 nm between the intercalating moiety (pyrene basis) and the technetium binding moiety of these molecules. We found a direct relationship between the DNA damage, the distance of the radionuclide ([^99m^Tc]**Ia** and [^99m^Tc]**Ib** vs. [^99m^Tc]**II** and [^99m^Tc]**III**) to the DNA and the amount of activity (3–15 MBq). The direct damage caused by the short range Auger electrons of these pyrene complexes resulted in DSB as well as SSB. Recently, Chung et al. reported similar effects for [^99m^Tc]**Ia** with slight differences in the relation of DSB vs. SSB [2].

**Fig 7:**
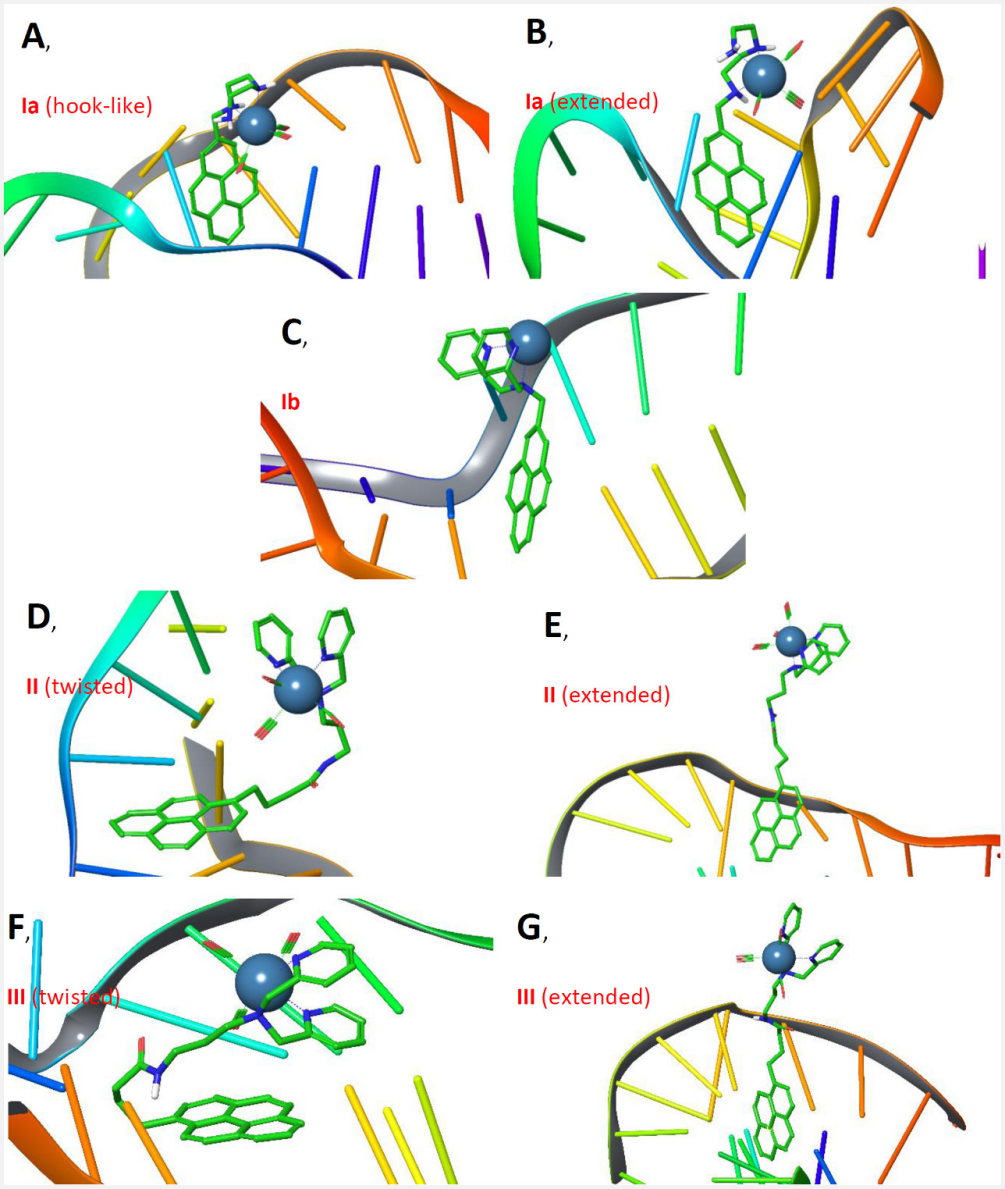
Examples of conformations of Tc-complexes Ia, Ib, II, and III in presence of DNA. (A) Ia, hook-like conformation, (B) Ia, extended conformation, (C) Ib, (D) II, twisted conformation, (E) II, extended conformation, (F) III, twisted conformation, (G) III, extended conformation.

^99m^TcO_4_^–^ behaves as a non-DNA-bonding molecule. Concerning the plasmid DNA damage caused by ^99m^TcO_4_^–^ (3–15 MBq), we found that most DNA damages could be avoided by the additional incubation of 0.2 M DMSO, because it functions as a scavenger of reactive, radical oxygen species, especially hydroxyl radicals. Therefore, almost all of SSB and DSB by ^99m^TcO_4_^–^ were shown to be indirect DNA damage caused by radiolysis of water.

The proximity between the Tc-core and the pyrene residue is not only dependent on the chain length but also on the conformation of the derivative itself. Thus, we predicted that both the structural change as well as the conformation could alter the distance between the Tc-core and the pyrene moiety; effectively placing the Tc in close proximity to the pyrene even with longer spacers. With this in mind, 100-ns molecular dynamics (MD) simulations were performed to get conformational sampling of the Tc-pyrene derivatives **Ia**, **Ib**, **II**, and **III** in the absence and presence of DNA. Details of the methodology and analysis of results for these simulations are included in the Supporting Information (S1 File). In broad terms, distances between pyrene moiety and Tc-core for **Ia** and **Ib** took short values (as expected) and **II** and **III** were in extended and twisted conformations. It is noteworthy, that **Ib** was very rigid due to the combination of DPA characteristics with a very short spacer (Tc-pyrene distances are around 7.5 Å in the absence and presence of DNA). Conversely, **Ia** adopted two sets of conformations when DNA is present due to the higher mobility of diethylenetriamine chelator exhibiting Tc-pyrene distances around 5.5 and 6.8 Å. Furthermore, the extended conformations were preferred over the twisted ones for **II** and **III** when DNA is present, see Fig 7. Comparing the proximities of the Tc-core and DNA as calculated by MD simulations, a closer proximity was calculated for **Ia**, followed by **Ib**, with **II** and **III** being further away.

For the tested ^99m^Tc-labeled pyrene derivatives, we observed that the type of DNA damage was strongly dependent on the distance between the DNA and the decaying radionuclide ^99m^Tc. By using pyrenes as a basis for intercalating molecules, we expected a specific bonding of our ^99m^Tc-labeled pyrenes to the minor grooves of the DNA, but it must be noted that all the pyrene derivatives used also acted as free radical scavengers and prevented indirect DNA damage themselves (Table 4).

If ^99m^Tc is located in immediate distance to the DNA as with complexes [^99m^Tc]**Ia** and [^99m^Tc]**Ib**, a higher yield of DSB was observed. However, a difference between [^99m^Tc]**Ia** and [^99m^Tc]**Ib** was still detectable. Derivative [^99m^Tc]**Ib** was more effective in inducing DSB. Differences between **Ia** and **Ib** according to the Tc-pyrene distances obtained by using MD simulations could explain different effects on DNA. **Ia** contains the ^99m^Tc more proximal to DNA, but ^99m^Tc of **Ib** is less moved.

Furthermore, the DNA damage could only slightly be reduced by incubation of the radical scavenger DMSO for [^99m^Tc]**Ib** while more than 50% of SSB was reduced for [^99m^Tc]**Ia** (Table 3). This is in striking contrast to the DNA damage caused by ^99m^TcO_4_^–^, which was reduced by greater than 80%. In spite of this scavenging effect, a respectable amount of direct SSB and DSB was still observed, suggesting that direct interactions were predominant between [^99m^Tc]**Ia**, [^99m^Tc]**Ib** and the DNA.

However, the different behavior of [^99m^Tc]**Ia** and [^99m^Tc]**Ib** was also dependent on the selected chelating unit for the ^99m^Tc(CO)_3_-core. MD simulations gave also some evidences of the effects of these chelators on distances between Tc and pyrene for **Ia** and **Ib**, because **Ia** has a shorter distance than **Ib**. However, **Ia** has also conformations with a distance similar to **Ib**, but **Ib** is more rigid than **Ia**. Recently, Chung et al. reported similar effects for [^99m^Tc]**Ia** with slightly differences in the relation of DSB vs. ESB [2]. In contrast to our recent studies they reported that the majority of DNA damages is caused by radiolysis of water. The chelating unit of [^99m^Tc]**Ia** consists of an aryl amine moiety whereas the chelating moiety of [^99m^Tc]**Ib** consists of two connected pyridine residues. Thus, there might be the possibility of a scavenging effect induced by the DPA unit.

The plasmids were incubated with ^99m^Tc-labeled pyrenes of high specific activity without an excess of unlabeled chelators. The maximum incubated ^99m^Tc activity of 15 MBq resulted in 4.7·10^11^ atoms of ^99m^Tc and approx. 10·10^11^ atoms of long-lived ^99^Tc. An amount of 200 ng of pUC 19 plasmid DNA corresponds to 6.88·10^10^ plasmid molecules. Normally, pyrene molecules bind to double-stranded DNA in the minor groove. The distance between two minor grooves is determined to be 10 base pairs. Therefore, we assumed that there are 270 binding sites per plasmid for ^99m^Tc-labeled pyrene derivatives. For our labeling procedure, approx. seven ^99m^Tc-labeled pyrene molecules (plus approx. fourteen ^99^Tc-labeled pyrene molecules) per plasmid corresponded to 15 MBq of activity in one assay. However, this result demonstrated that every available pyrene molecule could be bound to plasmid DNA.

This activity dependent DNA damage was shown to be caused by the emitted Auger electrons and not by the long-distance γ-rays of ^99m^Tc. This result indicated that the impact of the Auger events of [^99m^Tc]**Ia** and [^99m^Tc]**Ib** was occurred either direct or indirect with an extremely high local energy transfer and could not be prevented by DMSO or other radical scavengers like the pyrenes themselves. SSB are usually assumed to be radical-mediated. However, we demonstrated that the induction of SSB by DNA-bound pyrenes [^99m^Tc]**Ia** and [^99m^Tc]**Ib** might occur via another mechanism. It is possible that these SSB were also caused by direct interactions of the low-energy electrons emitted and plasmid-bound ^99m^Tc-pyrene derivatives could merely damage a single strand as assumed by Kotzerke et al. [16]. On average, 4.9 electrons of ^99m^Tc are emitted per decay. Those events create a dramatic charge transfer in the electron shell, which can cause a Coulomb explosion of the molecule, as discussed by Pomplun and Sutmann [28].

By comparing our studies with Balagurumoorthy’s results, several differences emerge. They synthesized a series of ^125^I-labeled Hoechst derivatives with a minor groove-binding motif and an increasing distance between the radiolabeled molecule and the binding position (ranging from 10.5 Å to 13.9 Å) [3]. The DSB yield decreased with distance from 0.50 DSB/decay to 0.00 DSB/decay, demonstrating the crucial influence of relatively small distances. Although DMSO could not prevent the DSB induced damage at the closest distance, their occurrence was reduced (approx. 10-fold) at higher distances. Comparing these results with our results in Table 3, a lower yield of DSB is found in our experiments. The difference (0.044 vs. 0.50 DSB per plasmid and decay) is explainable by comparing the different characteristics of the emitted Auger electrons of ^99m^Tc and ^125^I. A commonly used DNA structure model assumes that the diameter of the DNA helix is 2 nm. Therefore, we concluded that the difference arises from Auger electrons which have a range lower than 2 nm. Comparing the spectra of ^99m^Tc and ^125^I, there are two relevant Auger electrons per decay for ^99m^Tc but 18 for ^125^I [1, 29]. Therefore, it is conclusive that ^125^I can cause direct DSB in a higher efficiency than ^99m^Tc. Regarding the maximum distance for an effect caused by Auger electrons, we found a value of 1.3 nm for ^99m^Tc, which is similar to that found by Balagurumoorthy [3].

## Conclusion

In this study, four new ^99m^Tc-labeled pyrene derivatives were developed and their direct DNA damages were quantified for both DSB and SSB using pUC 19 plasmid DNA as a model. It is evident that ^99m^Tc can develop radical-mediated DNA damage and pyrene is a known DNA intercalating molecule. We demonstrated that direct DSB as well as direct SSB to plasmid DNA occurred in high efficiency due to the emitted Auger electrons of ^99m^Tc when the radionuclide is located in close proximity ([^99m^Tc]**Ia** and [^99m^Tc]**Ib**). The result showed that this effect could not be prevented by DMSO, a free radical scavenger, providing further proof of the direct action of Auger electrons on DNA. For the first time, we were able to extinguish this effect for ^99m^Tc by increasing the distance of the pyrene moiety and the chelating unit for ^99m^Tc by using linkers with different carbon chain lengths.

In contrast to the DNA binding ^99m^Tc-labeled pyrene molecules, ^99m^TcO_4_^–^ was applied as a non-DNA binding molecule in our experiments, since nearly the whole DNA damage caused by ^99m^TcO_4_^–^ is preventable by incubating DMSO.

In summary, it was shown that Auger electrons from ^99m^Tc can destroy DNA by direct interaction, however, only if the isotope is placed directly at the DNA. Clinical relevant Auger electron tumor therapy is hampered by the prerequisite of DNA binding which is hindered by cell and nucleus membranes. Vice versa this shield prevents critical damage in diagnostic application in nuclear medicine.

## Supporting Information

S1 File Syntheses of the pyrene chelators and details of the computational molecular dynamics calculations. (DOCX)
