# Supplementary material for: Direct and Auger Electron-Induced, Single- and Double-Strand Breaks on Plasmid DNA Caused by 99mTc-Labeled Pyrene Derivatives and the Effect of Bonding Distance
Source: PLoS One. 2016 Sep 1;11(9):e0161973. doi: 10.1371/journal.pone.0161973 (PMC5008623; doi:10.1371/journal.pone.0161973)
Supplement: S1 File — (DOCX) [file pone.0161973.s001.docx]

**S1 File. Syntheses of the pyrene chelators and details of the computational molecular dynamics calculations.**

**Supporting information for**

**Direct and Auger electron-induced, single- and double-strand breaks on plasmid DNA caused by ^99m^Tc-labeled pyrene derivatives and the effect of bonding distance**

Falco Reissig^1^, Constantin Mamat^2^, Joerg Steinbach^2^, Hans-Juergen Pietzsch^2^, Robert Freudenberg^1^, Carlos Navarro-Retamal^3^, Julio Caballero^3^, Joerg Kotzerke^1^, Gerd Wunderlich^1*^

^1^ University Hospital/ Faculty of Medicine Carl Gustav Carus, Technische Universität Dresden, Department of Nuclear Medicine, Dresden, Germany

^2^ Helmholtz-Zentrum Dresden-Rossendorf, Institute of Radiopharmaceutical Cancer Research, Dresden, Germany

^3^ Centro de Bioinformática y Simulación Molecular, Facultad de Ingeniería, Universidad de Talca, 2 Norte 685, Casilla 721, Talca, Chile

**Content:**

General remarks on chemical and radiochemical syntheses ………………………………S2

Syntheses of the pyrene chelators …………………………………………………………S3

Preparation of tricarbonyl kits for ^99m^Tc-labeling …………………………………………S8

Preparation of the pyrene complexes [^99m^Tc]**Ia**, [^99m^Tc]**Ib**, [^99m^Tc]**II**, [^99m^Tc]**III** ………..S9

Preparation of the pyrene complexes [^99^Tc]**Ia**, [^99^Tc]**Ib**, [^99^Tc]**II**, [^99^Tc]**III** …………....S10

Details of the computational molecular dynamics (MD) calculations …………………..S11

Analysis of the W and D models ………………………………………………………...S13

**General**

NMR spectra were recorded on an Agilent DD-2 (400 MHz) with ProbeOne and chemical shifts of the ^1^H and ^13^C spectra were reported in parts per million (ppm) using the solvent shifts for ^1^H and ^13^C as internal standards. All melting points were determined on a Galen III (Cambridge Instruments) melting point apparatus (Leica, Vienna, Austria) and are uncorrected. Anhydrous solvents (methanol, acetonitrile) were purchased from Sigma Aldrich (anhydrous, over molecular sieves, 99.7%; Schnelldorf, Germany) and other chemicals used for the syntheses were purchased from commercial suppliers and used as received. Chromatographic separations and TLC detections were carried out with Merck Silica Gel 60 (63–200 μm) and Merck Silica Gel 60 F_254_ sheets (Merck, Darmstadt), respectively. TLCs were developed by visualization under UV light (λ = 254 nm). Most relevant emissions of ^99m^Tc listed in Table A.

**Table A: Overview of the most relevant emissions of ^99m^Tc. Data were obtained from Howell [**[**1**](#_ENREF_1)**]. The selection of the listed emissions was based on the emission probability and the dose contribution. (IC: electrons from internal conversion, CK: Coster-Kronig electrons)**

| **Nomenclature** | **Average energy (keV)** | **Yield per decay (%)** | **Range (μm)** |
| --- | --- | --- | --- |
| Gamma2 | 141 | 88.9 |  |
| IC 1 M,N | 1.82 | 99.1 | 0.165 |
| IC 2 K | 119 | 8.43 | 193 |
| Auger KLL | 15.3 | 1.26 | 5.57 |
| Auger LMM | 2.05 | 8.68 | 0.199 |
| CK MMX | 0.116 | 74.7 | 0.006 |
| Auger MXY | 0.226 | 110 | 0.011 |
| CK NNX | 0.033 | 198 | 0.002 |

**Synthesis of the pyrene chelators**

All reactions concerning the reduction procedure with NaBH_4_ were carried out under an argon atmosphere. Pyrene **9** was synthesized according to Hafliger et al. [[12](#_ENREF_12)], compound **6a** was prepared according to Pretze and Mamat [[23](#_ENREF_23)] and **6b** was prepared according to Adres-Guisot et al. [[24](#_ENREF_24)].

Reaction path to the DPA-containing pyrene **4**.

*1-(Pyren-1-yl)-N-(pyridin-2-ylmethyl)methanamine****(3)****:*

A mixture of 1‑pyrenecarboxaldehyde (**2**, 500 mg, 2.17 mmol) and 2-picolylamine (**1**, 470 mg, 4.34 mmol) in anhydrous methanol (40 mL) was heated under reflux for 3 h under an argon atmosphere. Afterwards, NaBH_4_ (656 mg, 17.0 mmol) was added in portions. Then, the solution was stirred at room temperature for another 3 h and the solvent was evaporated to 1/3 of its original volume. Water (10 mL) and NaHCO_3_ (700 mg) were added to the concentrated solution, which was then stirred for 10 min at rt, and the aqueous layer was extracted with chloroform (3 x 25 mL). The combined organic layers were dried over Na_2_SO_4_ and the solvent was evaporated. The residue was purified by column chromatography on silica gel (CHCl_3_/MeOH, 96/4, v/v) to give **3** as yellow oil in 90% yield (630 mg). ^1^H-NMR (400 MHz, CDCl_3_): δ = 4.09 (s, 2H, CH_2_), 4.55 (s, 2H, CH_2_Ar), 7.17 (dt, ^3^*J* = 5.0 Hz, ^3^*J* = 7.6 Hz, 2H, H-5), 7.49 (d, ^3^*J* = 7.7 Hz, 2H, H-3), 7.60 (dt, ^4^*J* = 1.8 Hz, ^3^*J* = 7.6 Hz, 2H, H-4), 8.00 (t, ^3^*J* = 7.7 Hz, 1H, H_Ar_), 8.02–8.20 (m, 7H, H_Ar_), 8.40 (d, ^3^*J* = 9.5 Hz, 1H, H_Ar_), 8.60 (br. d, ^3^*J* = 5.0 Hz, 2H, H-6) ppm. ^13^C-NMR (101 MHz, CDCl_3_): δ = 51.5, 55.2 (2 x CH_2_), 122.1 (C-5), 122.6 (C-3), 123.5 (C-H_Ar_), 124.8 (C-H_Ar_), 124.x (C-q), 125.1 (C-H_Ar_), 125.2 (C-H_Ar_), 126.0 (C-H_Ar_), 127.2 (C-H_Ar_), 127.3 (C-H_Ar_), 127.6 (C-H_Ar_), 127.7 (C-H_Ar_), 129.3 (C-q), 130.8 (C-q), 131.0 (C-q), 131.5 (C-q), 133.8 (C-q), 136.6 (C-4), 149.8 (C-6), 156.0 (C-2).

*1-(Pyren-1-yl)-N,N-bis(pyridin-2-ylmethyl)methanamine****(4)****:*

2-(chloromethyl)pyridine hydrochloride (356 mg, 2.17 mmol) and K_2_CO_3_ (600 mg, 4.34 mmol) were added to a solution of **3** (700 mg, 2.17 mmol) in anhydrous acetonitrile (60 mL). The mixture was then heated under reflux for 6 h under an argon atmosphere. Reaction process was monitored by TLC. After the reaction was completed, the solvent was removed and the crude product was purified by column chromatography on silica gel (CHCl_3_/MeOH, 96/4, v/v) to give **4** as a pale yellow oil in 70% yield (628 mg). ^1^H-NMR (400 MHz, CDCl_3_): δ = 3.92 (s, 4H, CH_2_), 4.40 (s, 2H, CH_2_Ar), 7.12 (dt, ^3^*J* = 4.8 Hz, ^3^*J* = 6.0 Hz, 2H, H-5), 7.49 (d, ^3^*J* = 8.0 Hz, 2H, H-3), 7.60 (dt, ^4^*J* = 1.4 Hz, ^3^*J* = 8.0 Hz, 2H, H-4), 7.96–8.20 (m, 8H, H_Ar_), 8.38 (d, ^3^*J* = 9.5 Hz, 1H, H_Ar_), 8.54 (br. d, ^3^*J* = 5.0 Hz, 2H, H-6) ppm. ^13^C-NMR (101 MHz, CDCl_3_): δ = 57.3, 60.7 (2 x CH_2_), 122.1 (C-5), 123.5 (C-3), 124.2 (C-H_Ar_), 124.6 (C-H_Ar_), 124.9 (C-q), 125.0 (C-H_Ar_), 125.1 (C-H_Ar_), 125.2 (C-q), 126.0 (C-H_Ar_), 127.1 (C-H_Ar_), 127.2 (C-H_Ar_), 127.6 (C-H_Ar_), 128.4 (C-H_Ar_), 130.0 (C-q), 130.9 (C-q), 131.0 (C-q), 131.4 (C-q), 132.7 (C-q), 136.4 (C-4), 149.0 (C-6), 159.8 (C-2).

Reaction path to the DPA-containing pyrenes **8a** and **8b**.

*3-Azido-N,N-bis(pyridin-2-ylmethyl)propan-1-amine* ***(7a)****:*

Di-(2-picolyl)amine (**5**, 300 mg, 1.51 mmol), 3-azidopropyl tosylate [[23](#_ENREF_23)] (**6a**, 461 mg, 1.81 mmol) and DIPEA (292 mg, 2.26 mmol) were dissolved in chloroform (10 mL) and the resulting mixture was heated to 60°C overnight. After cooling to rt and removal of the solvent, ethyl acetate (20 mL) and water (20 mL) was added, the aqueous layer extracted with ethyl acetate (3 x 20 mL), the combined organic layers dried over Na_2_SO_4_ and the solvent removed. The crude product was purified via column chromatography to give **7a** (250 mg, 59%) as pale yellow syrup. ^1^H-NMR (400 MHz, CDCl_3_): δ = 1.75-1.83 (m, 2H, CH_2_), 2.64 (t, ^3^*J* = 7.0 Hz, 2H, CH_2_N), 3.30 (t, ^3^*J* = 7.0 Hz, 2H, CH_2_N_3_), 3.82 (s, 4H, CH_2_Py), 7.15 (dt, ^3^*J* = 4.8 Hz, ^3^*J* = 7.5 Hz, 2H, H-5), 7.48 (d, ^3^*J* = 7.8 Hz, 2H, H-3), 7.66 (dt, ^4^*J* = 1.8 Hz, ^3^*J* = 7.5 Hz, 2H, H-4), 8.53 (dd, ^4^*J* = 1.8 Hz, ^3^*J* = 4.8 Hz, 2H, H-6) ppm. ^13^C-NMR (101 MHz, CDCl_3_): δ = 26.7 (CH_2_), 49.6 (CH_2_N_3_), 51.4 (CH_2_N), 60.7 (CH_2_Py), 122.2 (C-3), 123.1 (C-5), 136.5 (C-4), 149.2 (C-6), 159.6 (C-2) ppm. MS (ESI+) *m/z* = 305 (10) [M+Na]^+^, 283 (100) [M+H]^+^.

*6-Azido-N,N-bis(pyridin-2-ylmethyl)hexan-1-amine* ***(7b)****:*

Di-(2-picolyl)amine (**5**, 334 mg, 1.68 mmol), 6-azidohexyl tosylate [[24](#_ENREF_24)] (**6b**, 598 mg, 2.01 mmol) and DIPEA (542 mg, 4.19 mmol) were dissolved in chloroform (10 mL) and the resulting mixture was heated to 60°C overnight. After cooling to rt and removal of the solvent, ethyl acetate (20 mL) and water (20 mL) was added, the aqueous layer extracted with ethyl acetate (3 x 20 mL), the combined organic layers dried over Na_2_SO_4_ and the solvent removed. The crude product was purified via column chromatography to give **7b** (299 mg, 55%) as pale yellow syrup. ^1^H-NMR (400 MHz, CDCl_3_): δ = 1.24–1.33 (m, 2H, CH_2_), 1.49–1.58 (m, 2H, CH_2_), 2.54 (t, ^3^*J* = Hz, 2H, CH_2_N), 3.21 (t, ^3^*J* = Hz, 2H, CH_2_N_3_), 3.80 (s, 4H, CH_2_Py), 7.14 (dt, ^3^*J* = 4.7 Hz, ^3^*J* = 7.4 Hz, 2H, H-5), 7.52 (d, ^3^*J* = 7.8 Hz, 2H, H-3), 7.65 (dt, ^4^*J* = 1.8 Hz, ^3^*J* = 7.8 Hz, 2H, H-4), 8.52 (dd, ^4^*J* = 1.8 Hz, ^3^*J* = 4.7 Hz, 2H, H-6) ppm. ^13^C-NMR (101 MHz, CDCl_3_): δ = 26.7, 27.0, 27.1, 28.9 (4 x CH_2_), 51.5 (CH_2_N_3_), 54.4 (CH_2_N), 60.9 (CH_2_Py), 122.0 (C-5), 123.0 (C-3), 136.5 (C-4), 149.1 (C-6), 160.2 (C-2). MS (ESI+): *m/z* = 325 (100) [M+H]^+^.

*N-(3-(bis(pyridin-2-ylmethyl)amino)propyl)-4-(pyren-1-yl)butanamide (****8a)****:*

Compound **7a** (100 mg, 0.35 mmol) and PPh_3_ (102 mg, 0.39 mmol) were dissolved in a mixture of acetonitrile and water (10/1 v/v) and the resulting mixture was heated to 60°C for 3.5 h. Next, 1-pyrenebutyric acid *N*-hydroxysuccinimide ester (150 mg, 0.39 mmol) was added and the mixture was stirred at ambient temperature for 3 h. Afterwards, the solvent was removed and the crude product was purified using automated column chromatography (Biotage Isolera Four, Uppsala, Sweden) to give **8a** (100 mg, 57%) as pale yellow syrup. ^1^H-NMR (400 MHz, CDCl_3_): δ = 1.66–1.75 (m, 2H, CH_2_), 2.17–2.28 (m, 2H, CH_2_), 2.35 (t, ^3^*J* = 7.4 Hz, 2H, CH_2_Ar), 2.59 (t, ^3^*J* = 6.0 Hz, 2H, CH_2_N), 3.32 (dt, ^3^*J* = 5.6 Hz, ^3^*J* = 5.8 Hz, 2H, CH_2_NH), 3.38 (t, ^3^*J* = 7.5 Hz, 2H, CH_2_C=O), 3.66 (s, 4H, CH_2_Py), 6.91 (dt, ^3^*J* = 5.1 Hz, ^3^*J* = 7.2 Hz, 2H, H-5), 7.24 (d, ^3^*J* = 7.6 Hz, 2H, H-3), 7.45 (dt, ^4^*J* = 1.8 Hz, ^3^*J* = 7.6 Hz, 2H, H-4), 7.63 (t, ^3^*J* = 5.5 Hz, 1H, NH), 7.84 (d, ^3^*J* = 7.9 Hz, 1H, H_Ar_), 7.94–8.01 (m, 3H, H_Ar_), 8.04 (d, ^3^*J* = 3.9 Hz, 1H, H_Ar_), 8.06 (d, ^3^*J* = 2.4 Hz, 1H, H_Ar_), 8.14 (d, ^3^*J* = 2.4 Hz, 2H, H_Ar_), 8.26 (d, ^3^*J* = 9.4 Hz, 1H, H_Ar_), 8.30 (br. d, ^3^*J* = 5.1 Hz, 2H, H-6). ^13^C-NMR (101 MHz, CDCl_3_): δ = 26.3, 27.7, 33.0, 35.9, 38.5 (5 x CH_2_), 53.3 (CH_2_N), 60.0 (CH_2_Py), 122.2 (C-5), 123.4 (C-3), 123.6 (C-H_Ar_), 124.7 (C-H_Ar_), 124.8 (C-H_Ar_), 124.9 (C-H_Ar_), 125.0 (C-q), 125.1 (C-q), 125.9 (C-H_Ar_), 126.7 (C-H_Ar_), 127.3 (C-H_Ar_), 127.4(C-H_Ar_), 127.6 (C-H_Ar_), 128.9 (C-q), 129.9 (C-q), 131.0 (C-q), 131.5 (C-q), 136.3 (C-q), 136.5 (C-4), 148.9 (C-6), 159.1 (C-2), 172.9 (C=O). MS (ESI+): *m/z* = 527 (100) [M+H]^+^.

*N-(6-(bis(pyridin-2-ylmethyl)amino)hexyl)-4-(pyren-1-yl)butanamide (****8b)****:*

Compound **7b** (100 mg, 0.31 mmol) and PPh_3_ (89 mg, 0.34 mmol) were dissolved in a mixture of acetonitrile and water (5 mL, 10/1 v/v) and the resulting mixture was heated to 60°C for 3.5 h. Next, 1-pyrenebutyric acid *N*-hydroxysuccinimide ester (131 mg, 0.34 mmol) was added and the mixture was stirred at ambient temperature for 3 h. Afterwards, the solvent was removed and the crude product was purified using automated column chromatography to give **8b** (114 mg, 61%) as pale yellow syrup. ^1^H-NMR (400 MHz, CDCl_3_): δ = 1.13–1.27 (m, 4H, CH_2_), 1.33–1.53 (m, 4H, CH_2_), 2.13–2.24 (m, 4H, CH_2_), 2.49 (t, ^3^*J* = 7.3 Hz, 2H, CH_2_N), 3.16 (dt, ^3^*J* = 5.5 Hz, ^3^*J* = 7.0 Hz, 2H, CH_2_NH), 3.36 (t, ^3^*J* = 7.1 Hz, 2H, CH_2_C=O), 3.76 (s, 4H, CH_2_Py), 5.57 (t, ^3^*J* = 5.5 Hz, 1H, NH), 6.91 (dt, ^3^*J* = 5.0 Hz, ^3^*J* = 7.5 Hz, 2H, H-5), 7.46 (d, ^3^*J* = 7.7 Hz, 2H, H-3), 7.59 (dt, ^4^*J* = 1.7 Hz, ^3^*J* = 7.7 Hz, 2H, H-4), 7.82 (d, ^3^*J* = 7.7 Hz, 1H, H_Ar_), 7.94–8.01 (m, 3H, H_Ar_), 8.04–8.10 (m, 2H, H_Ar_), 8.14 (d, ^3^*J* = 7.7 Hz, 2H, H_Ar_), 8.27 (d, ^3^*J* = 9.5 Hz, 1H, H_Ar_), 8.30 (br. d, ^3^*J* = 5.0 Hz, 2H, H-6). ^13^C-NMR (101 MHz, CDCl_3_): δ = 26.7, 26.8, 26.9, 27.6, 29.6, 32.9, 36.2, 39.5 (8 x CH_2_), 54.3 (CH_2_N), 60.4 (CH_2_Py), 122.0 (C-5), 123.1 (C-3), 123.5 (C-H_Ar_), 124.9 (C-H_Ar_), 125.0 (C-H_Ar_), 125.1 (C-q), 125.9 (C-H_Ar_), 126.7 (C-H_Ar_), 127.3 (C-H_Ar_), 127.4(C-H_Ar_), 127.6 (C-H_Ar_), 128.9 (C-q), 129.9 (C-q), 131.0 (C-q), 131.5 (C-q), 136.3 (C-q), 136.5 (C-4), 148.9 (C-6), 159.1 (C-2), 172.9 (C=O). MS (ESI+): *m/z* = 569 (100) [M+H]^+^.

**Preparation of tricarbonyl kits for ^99m^Tc-labeling**

Tricarbonyl kits for radiolabeling were prepared in-house. Every tricarbonyl kit consisted of the following components: 17 mg sodium tartrate, 3.5 mg sodium borate, 3.2 mg sodium carbonate and 8.1 mg potassium boranocarbonate. All kits were prepared by pipetting a stock solution into glass vials. Then, the vials were freeze-dried at 0.1 mbar vacuum and capped under protective nitrogen atmosphere.

**Preparation of the pyrene complexes [^99m^Tc]Ia, [^99m^Tc]Ib, [^99m^Tc]II, [^99m^Tc]III**

^99m^Tc was used as Na[^99m^Tc]TcO_4_ generator eluate obtained by elution from a ^99^Mo/^99m^Tc generator (Mallinckrodt Deutschland GmbH, Hennef, Germany) using a 0.9% NaCl solution. ^99m^TcO_4_^–^ (1 mL, ~ 5 GBq) was added to the tricarbonyl kit. Afterwards, the solution was mixed at 300 rpm for 30 min at 95°C and was used without further purification. The pH of the [^99m^Tc(CO)_3_(H_2_O)_3_]^+^ complex solution (~ 5 GBq/mL) was adjusted to 6-7 by adding PBS/1M HCl (190 µL, v/v, 1:1). Stock solutions of the pyrene derivatives **4**, **8a**, **8b** and **9** were prepared (1 µg/µL in EtOH). For radiolabeling, 30 µL of the respective pyrene stock solution was added to the [^99m^Tc(CO)_3_(H_2_O)_3_]^+^ complex solution and the resulting mixture was heated at 95°C and 500 rpm for 60 min. The resulting radiotracer was analysed and purified via HPLC (Merck Hitachi, Darmstadt, Germany) using a Merck Millipore Chromolith® Performance RP-18e (100 x 4.6 mm) column for analysis and purification. Eluents are purified water (solvent A; 0.05% TFA; Merck, Darmstadt, Germany) and HPLC-grade acetonitrile (Solvent B; VWR, Darmstadt, Germany; 0.05% TFA) used as gradient from 95:5% solvent A to 5:95% solvent A within 11 minutes. After purification, all final products [^99m^Tc]**Ia**, [^99m^Tc]**Ib**, [^99m^Tc]**II**, [^99m^Tc]**III** exhibit a radiochemical purity of greater than 95% and were used for the plasmid DNA experiments.

**Preparation of the pyrene complexes [^99^Tc]Ia, [^99^Tc]Ib, [^99^Tc]II, [^99^Tc]III**

All ^99^Tc-labeled pyrene complexes [^99^Tc]**Ia**, [^99^Tc]**Ib**, [^99^Tc]**II** and [^99^Tc]**III** were produced by the afore mentioned standard radiolabeling procedure for the ^99m^Tc-labeled pyrene complexes. Then, a stock solution of 1 GBq/mL (as 0.9% NaCl solution) of the respective ^99m^Tc complex was maintained for 72 hours and decays to obtain the appropriate ^99^Tc‑complex.

**Details of the computational molecular dynamics (MD) calculations**

Tc-complexes used in this study.

Tc-pyrene derivatives (including Tc with a formal charge of +1) were sketched using Maestro Editor (Maestro, version 9.7, Schrödinger, LLC, New York, NY, 2014) and then molecular energy minimizations were done using the force field OPLS_2005 for 300 cycles or until the maximum atom displacement was less than 0.05 Å. Next, two optimization steps were performed at B3LYP-STO-3G* followed by MO6-2X-LACVP**[1] using the module Jaguar of Schrödinger (Jaguar, version 8.8, Schrödinger, LLC, New York, NY, 2015)[2] to correctly predict the 3D configuration of the molecules.

Two solvated molecular models were prepared for each compound (**Ia**, **Ib**, **II**, and **III**): models in absence of DNA (W models) and in presence of DNA (D models). W and D models were solvated using orthorhombic boxes, with a distance of 10 Å in each axis, using the SPC water model [3].

The W models (WIa, WIb, WII, and WIII) were constructed by embedding 3D configurations of compounds **Ia**, **Ib**, **II**, and **III** in a water box. The D models (DIa, DIb, DII, and DIII) were constructed by preparing complexes between DNA and compounds **Ia**, **Ib**, **II**, and **III** embedded in a water box. For preparing DNA complexes, compounds **Ia**, **Ib**, **II**, and **III** were placed near the double-helix DNA molecule forming pi-stacking interactions between the pyrene moiety and DNA. The complex models were performed to replicate the interaction between a pyrene group and DNA as shown in the crystallographic structure deposited in Protein Data Bank (PDB 1Y9H)[4]. For this, each pyrene ring was intercalated inside the minor groove edge, sandwiched between the flanking two base pairs. Finally, each DNA complex was embedded in a water box to construct the final D models.

100-ns molecular dynamic (MD) simulations were performed using Desmond [5–7] module for the eight solvated models to extract the available distances between the technetium and the center of mass of pyrene for compounds **Ia**, **Ib**, **II**, and, **III** in the absence and presence of DNA. Prior to the production MD, a series of short MD using different ensembles at low and high temperatures were performed to equilibrate the system. In the production run, an NPT ensemble was used to maintain the integrity of the system, where The Nose-Hoover chain thermostat and the Martyna-Tobias-Klein barostat were used at 300K (relaxation time of 1ps) and 1 atm (relaxation time of 2ps). Also, a cutoff of 9 Å was used for the electrostatic and VDW interactions, where a smooth particle mesh, Ewald method, was used to describe properly electrostatic interactions. A time step of 2 fs was used to describe the motions of the molecules over time.

**Analysis of the W and D models**

MD trajectories were analyzed in order to get a sampling of the distances between Tc and the pyrene center of mass. Trajectories of the W models were used to identify possible bending and twisting of the derivatives without the presence of DNA. Figure S1a shows that the Tc-pyrene distances for **Ia** and **Ib** were 5.70 ± 0.23 Å and 7.49 ± 0.08 Å, respectively. It is noteworthy that **Ib** was very rigid due to the combination of DPA characteristics with the very short spacer. Conversely, **Ia** adopted a hook-like structure with Tc-pyrene distances varying over a higher range due to the higher mobility of diethylenetriamine chelator. The analysis of Tc-pyrene distances for **II** and **III** indicated that these derivatives are present in water medium in extended and twisted conformations (the twisted conformations are clearly preferred for **III**). Twisting was produced by the repulsion between hydrophobic groups of the spacer and the water molecules. After this analysis, it was clear that the definition of the Tc-pyrene distances must consider a conformational sampling of the molecular systems.

It was useful for us to consider the Tc-pyrene distances when DNA is present because of the intended medicinal application of these molecules. The trajectories of the D models are illustrated in Figure S1b. In broad terms, Tc-pyrene distances for **Ia** and **Ib** also exhibited short values and **II** and **III** were also in extended and twisted conformations when DNA is present, but some differences in the distribution of the conformations were observed. **Ib** maintained the rigid structure (Tc-pyrene distance = 7.51 ± 0.08 Å; example of conformation in Figure S2c), but **Ia** adopted two sets of conformations with Tc-pyrene distances of 5.52 ± 0.16 Å and 6.81 ± 0.19 Å. The first set was formed by the hook-like conformations similar to the ones found in water medium (Figure S2a), and the second set was formed by more extended conformations (Figure S2b). Furthermore, the distribution of the Tc-pyrene distances for **II** and **III** indicated that twisted conformations were less favored for **II** and **III** when DNA is present (examples of twisted and extended conformations for these compounds in the presence of DNA are in Figures S2d-g). Therefore, DNA caused a considerable reduction of twisted conformations; this fact means that Tc was separated from the DNA for compounds with longer spacers.

Radial distribution functions (RDFs) derived from trajectories of the D models were calculated to provide a more detailed analysis. RDFs were established between Tc and the whole DNA structure (Figure S3 shows the results). The relative intensities of the plots reflect the proximities of Tc from each derivative to the DNA structure considering the contribution of all conformations. The order of proximities between Tc and DNA according to RDF plots is **Ia** > **Ib** > **III** > **II**.

**Fig S1.** **Distances between Tc and pyrene center of mass for Ia, Ib, II, and III.** (A) in water medium, (B) in the presence of DNA.

A)


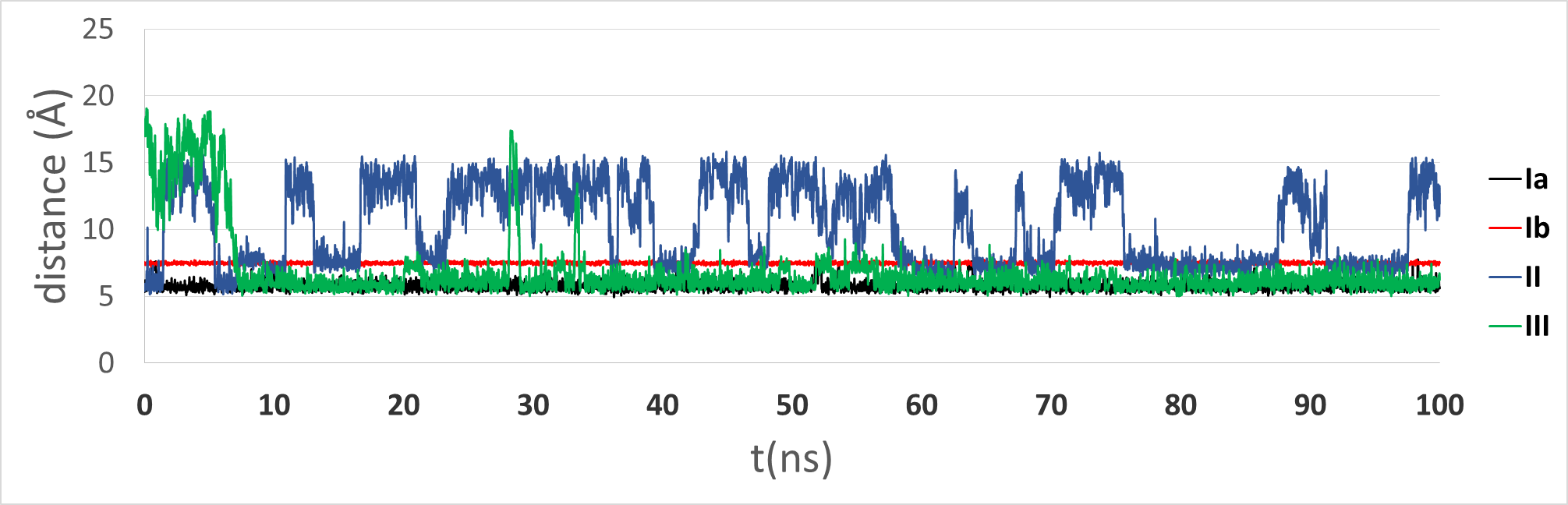


B)


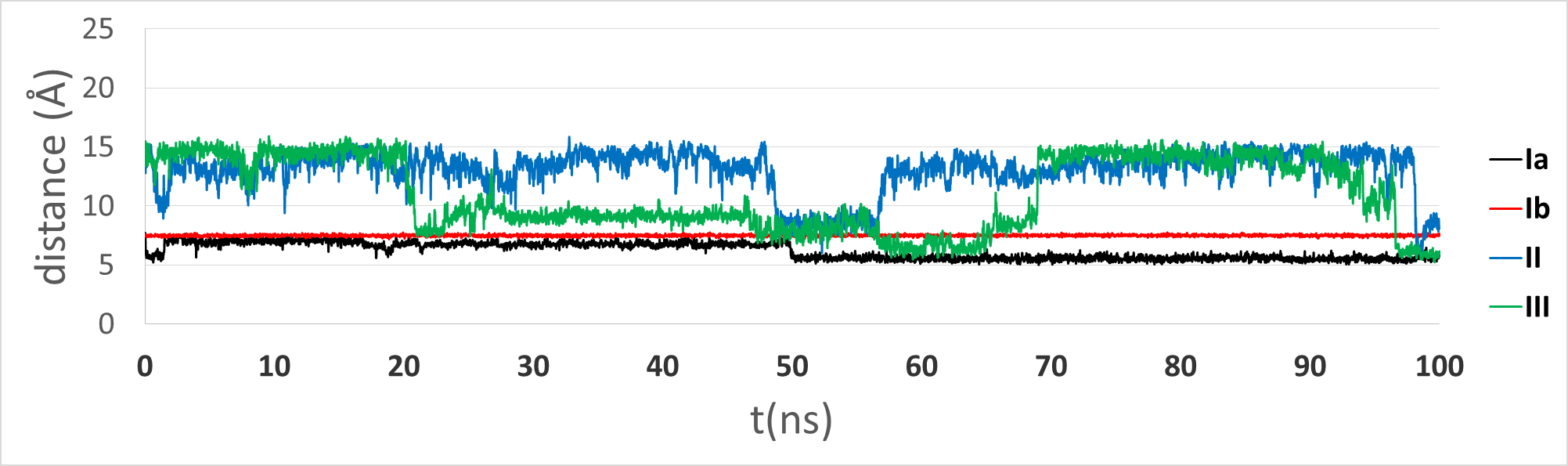


**Ia** in water medium (distance: 5.70 ± 0.23 Å).


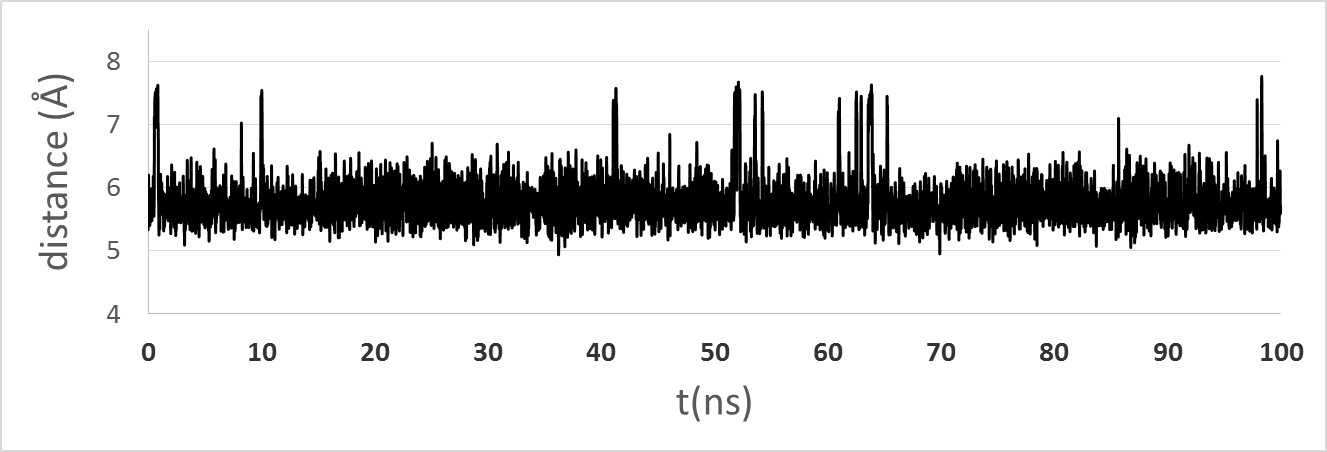


**Ib** in water medium (distance: 7.49 ± 0.08 Å).

**
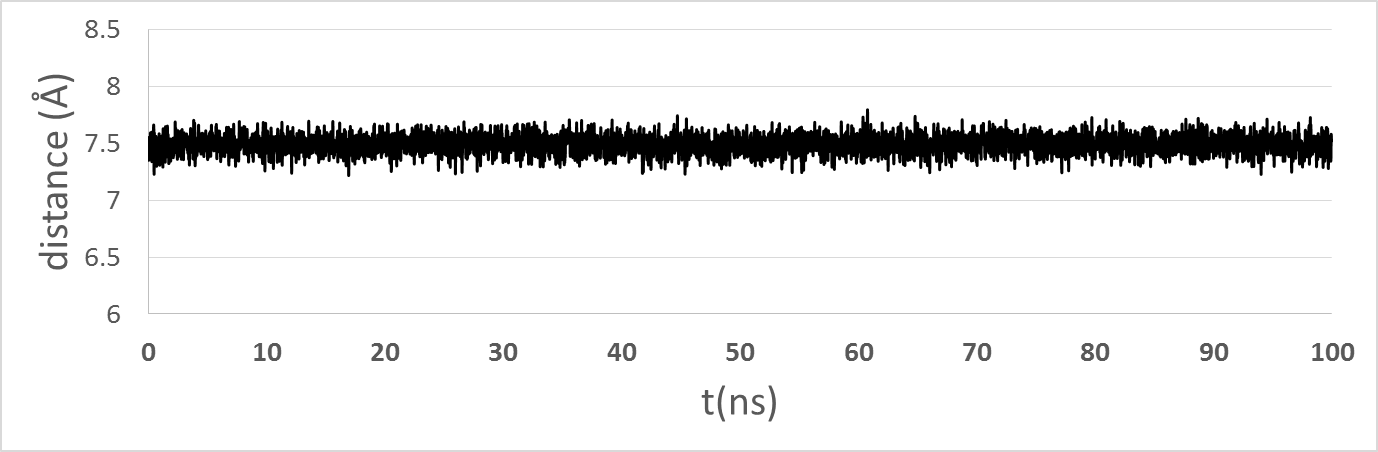
**

**Ib** and **Ia** together in water medium.


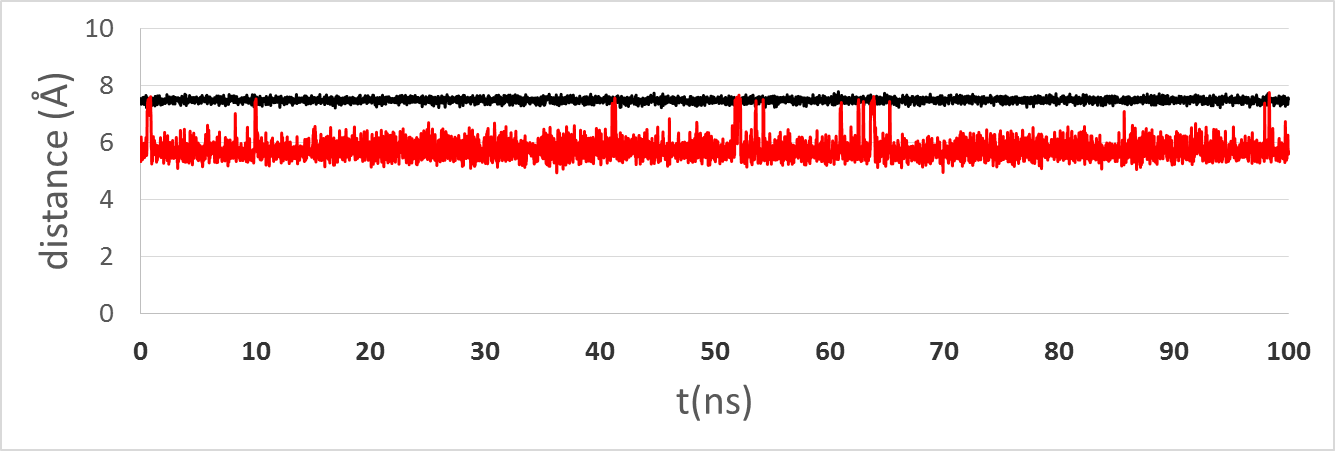

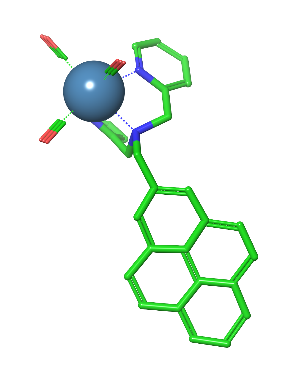

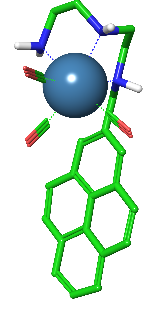

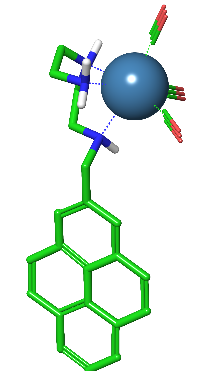


**Ia** has a shorter distance than **Ib**. However, **Ia** has also conformations with a distance similar to **Ib**, but **Ib** is more rigid than **Ia**.

**II** in water medium (more representative states: A) distance: 13.47 ± 0.88 Å, B) distance: 7.40 ± 0.38 Å, C) distance: 5.78 ± 0.12 Å.

**A**


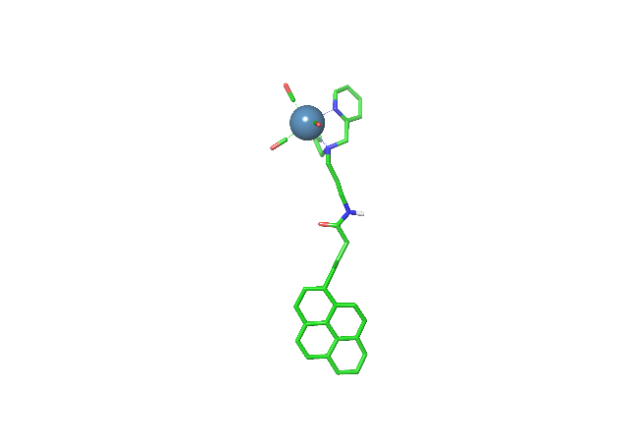

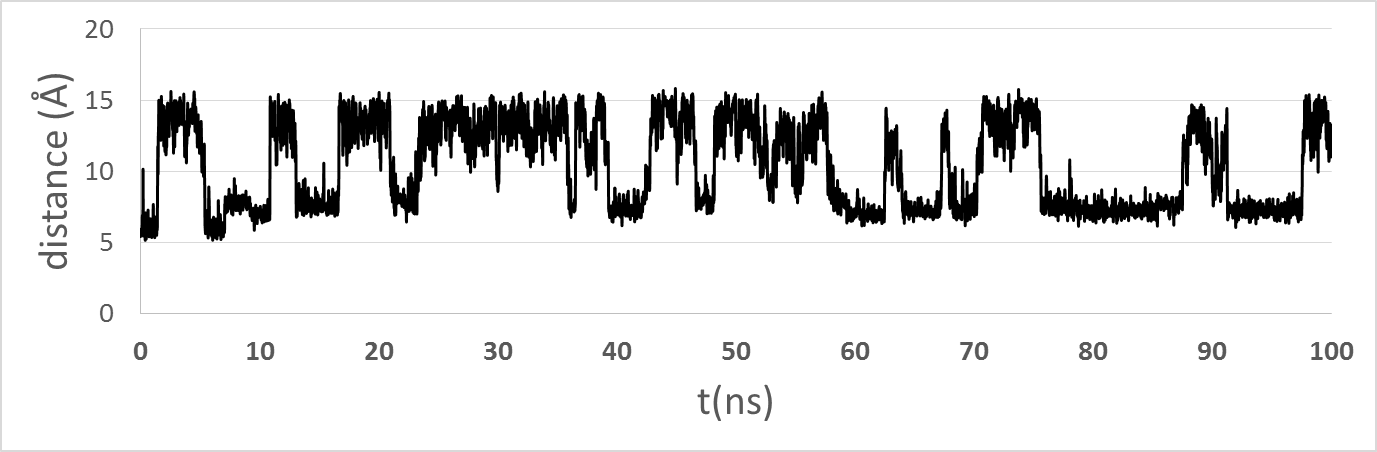


**C**

**B**


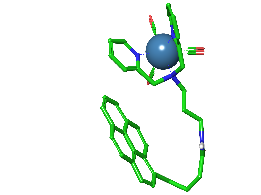

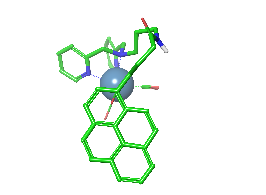


**III** in water medium (more representative states: distance: 5.69 ± 0.20 Å, There are no higher preferred distances.


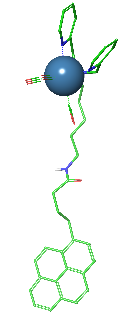

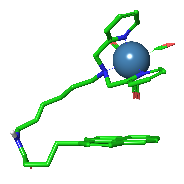

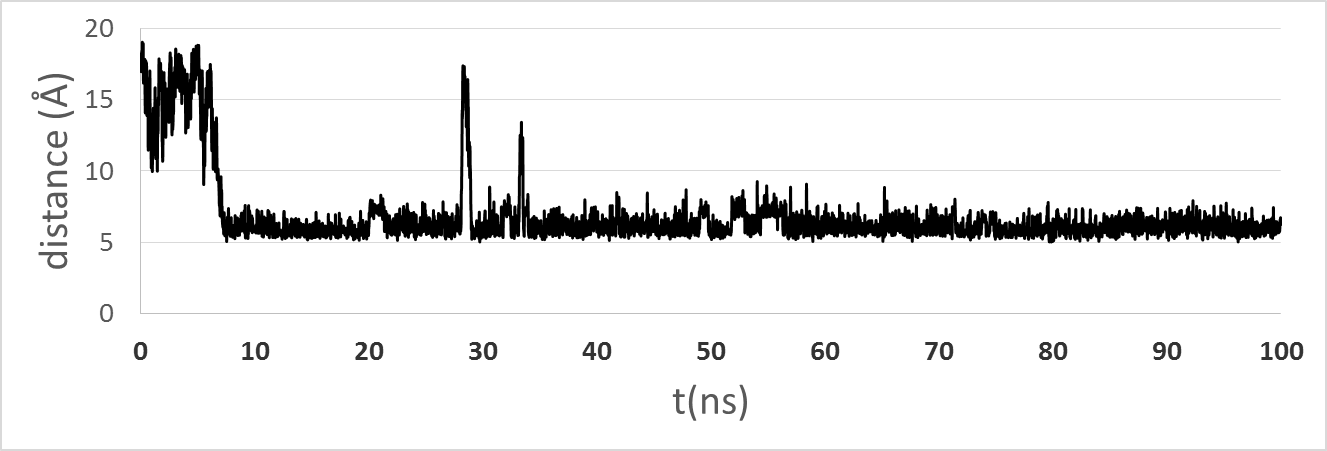


**III** (black) and **II** (red) together in water medium.

Comparisons compound **Ib** in water and in DNA-water. (water distance: 7.49 ± 0.08 Å, DNA distance: 7.51 ± 0.08 Å).


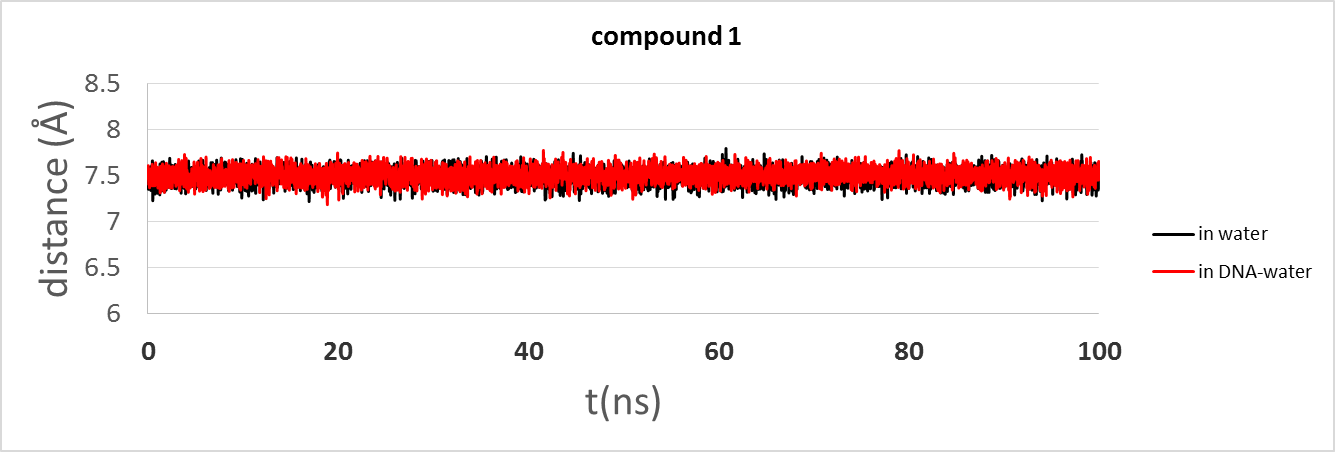


SSS

Very rigid. Distances in water and DNA-water are the same.


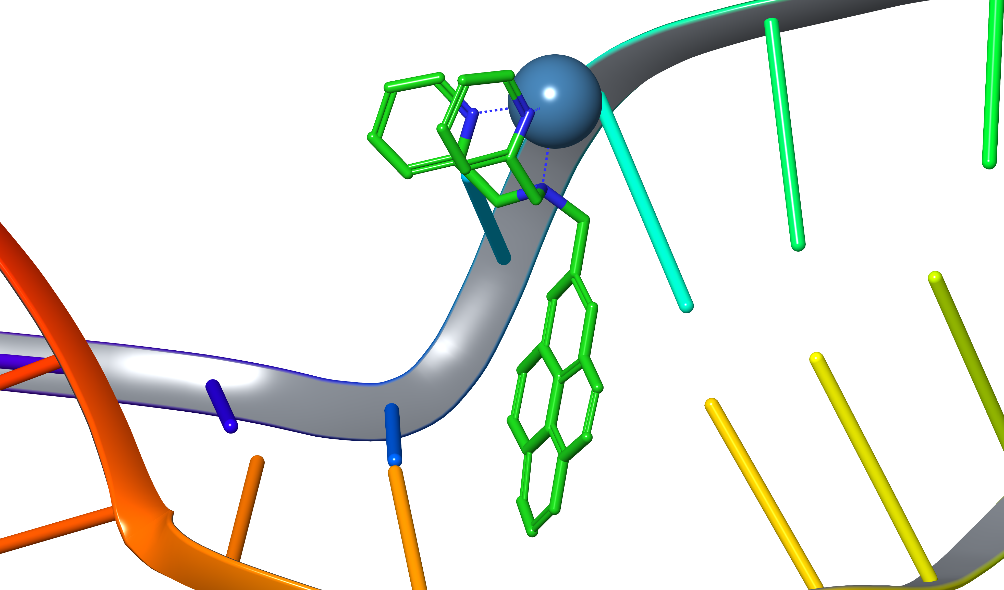


Comparison of compound **Ia** in water and in DNA-water (water distance: 5.70 ± 0.23 Å, DNA distances: 5.52 ± 0.16 Å and 6.81 ± 0.19 Å).


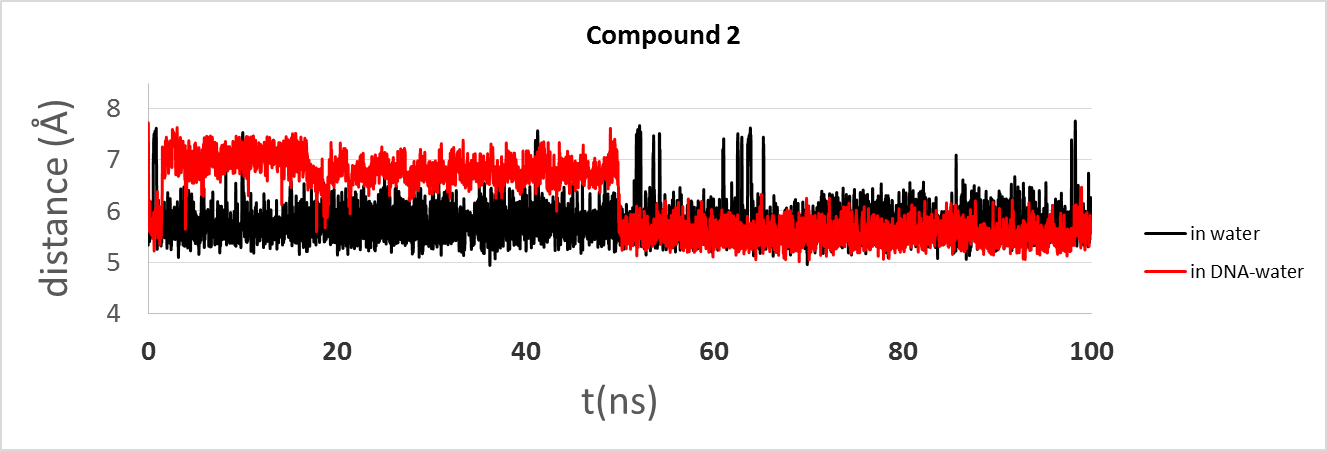


SSS

Two main clusters of conformations with distances 5.5 Å and 6.8 Å in DNA-water. Cluster 5.5 Å is more accessible when DNA is not present.


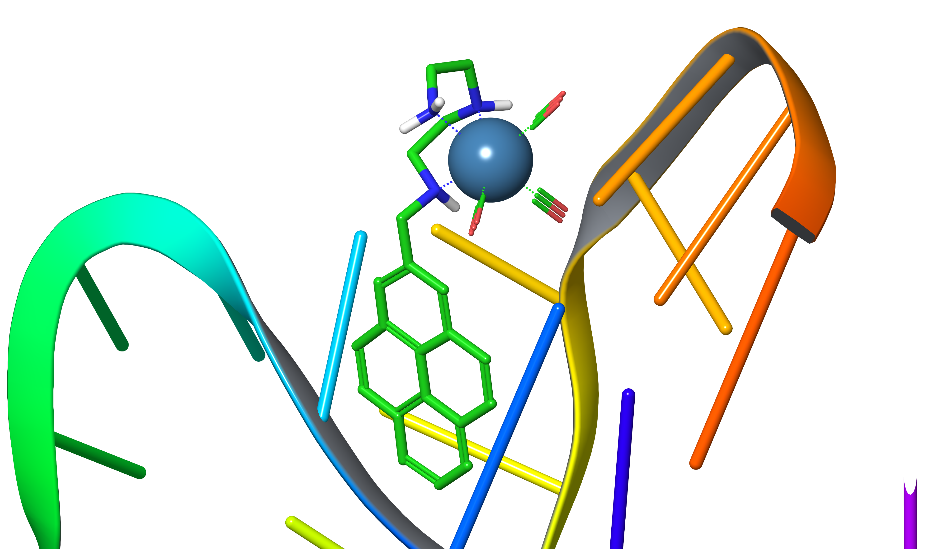

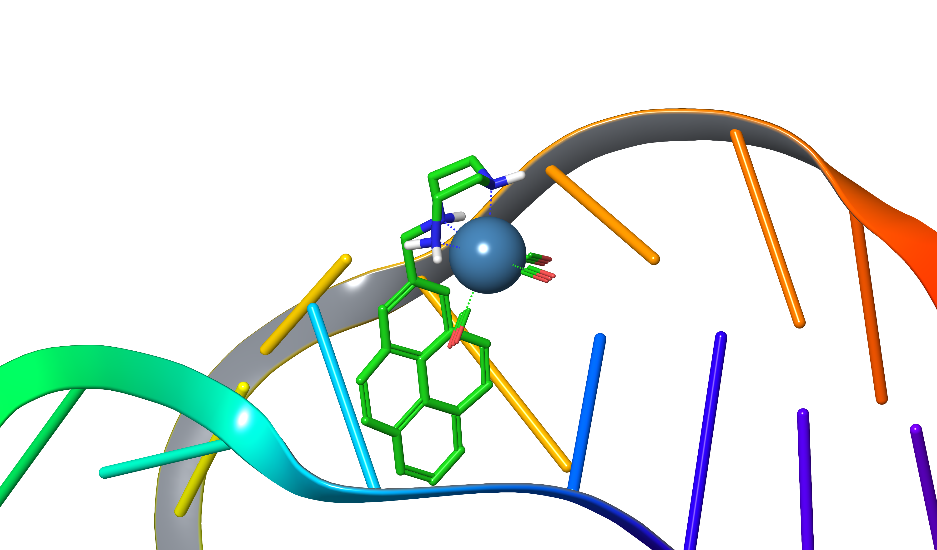
5.5 Å 6.8 Å

Comparison of compound **II** in water and in DNA-water (water distances: 13.47 ± 0.88 Å, 7.40 ± 0.38 Å, and 5.78 ± 0.12 Å; DNA distances: 13.61 ± 0.88 Å, 8.25 ± 0.18 Å)


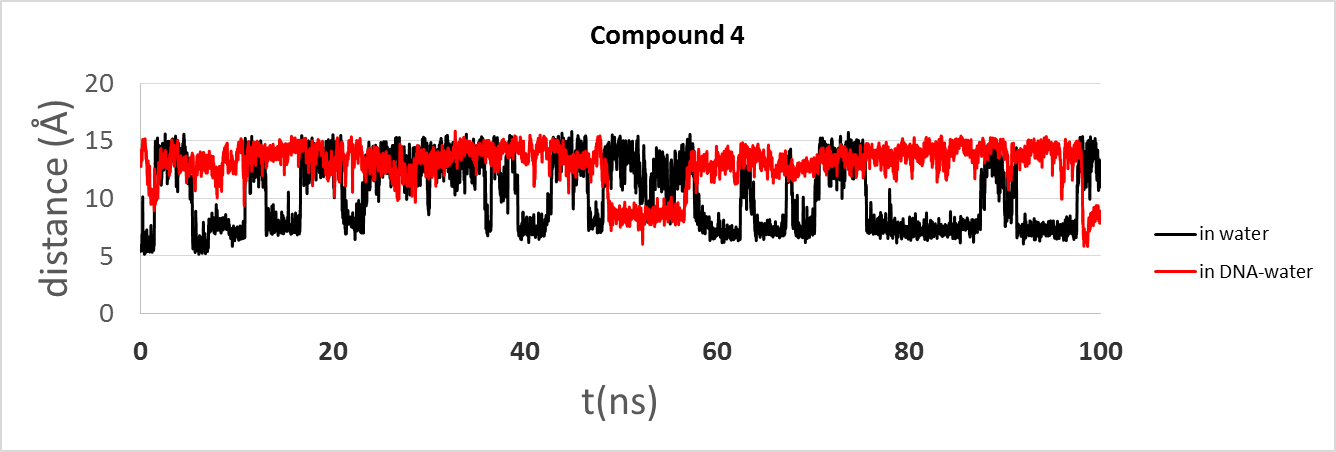


SSS

Two main clusters with 8 Å and 13.6 Å in DNA-water. Extended cluster is more probable.


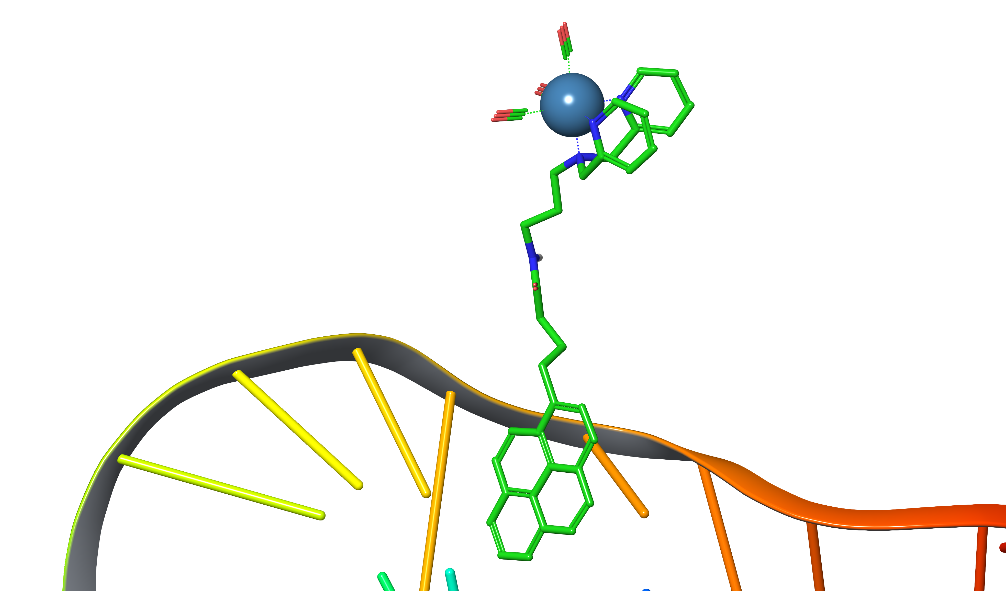

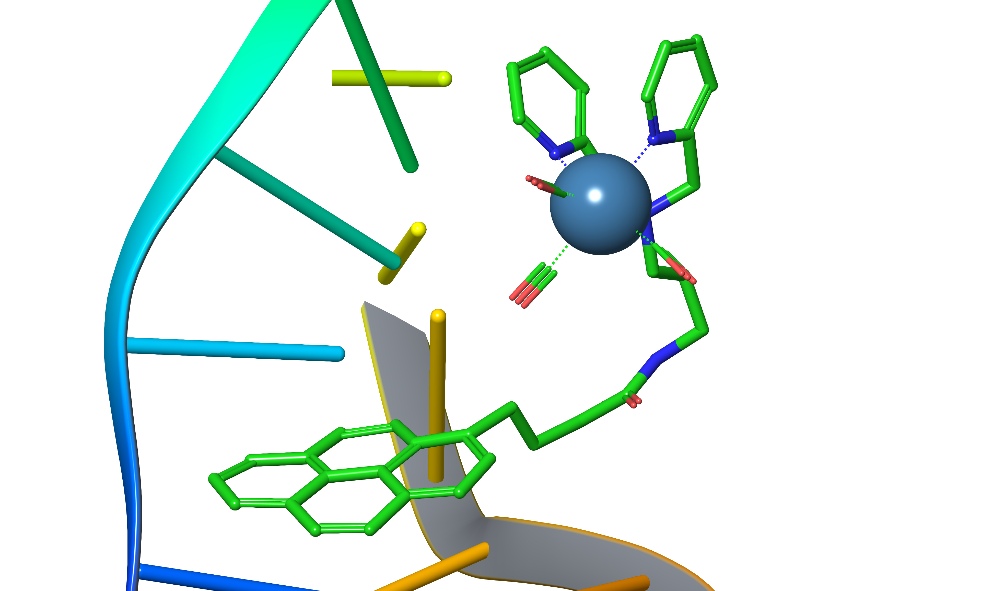
8 Å 13.6 Å

Comparison of compound **III** in water and in DNA-water (water distance: 5.69 ± 0.20 Å), (DNA distance: 9.14 ± 0.35 Å, 14.44 ± 0.46 Å, 6.13 ± 0.14 Å).


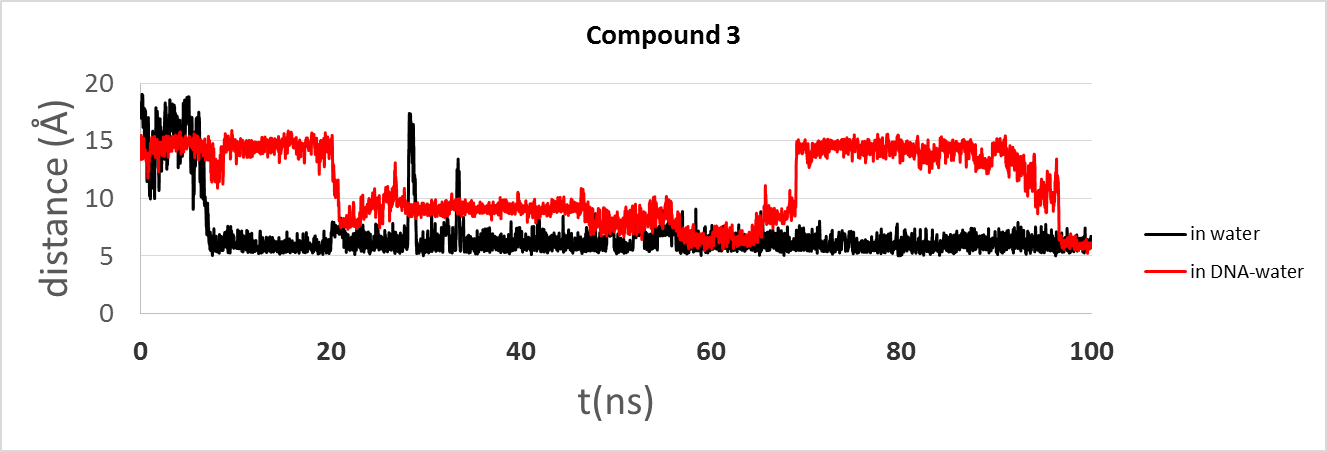


SSS

Three clusters with 6 Å, 9 Å and 14 Å in DNA-water. In water there is no defined distance when the structure is extended. It seems that two preferred clusters (around 9 Å and 14 Å) appear when DNA is included


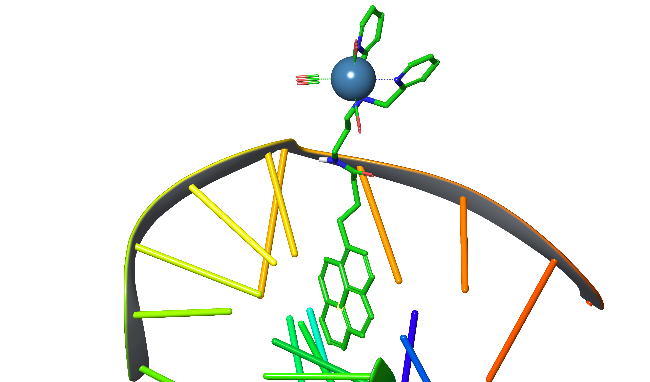

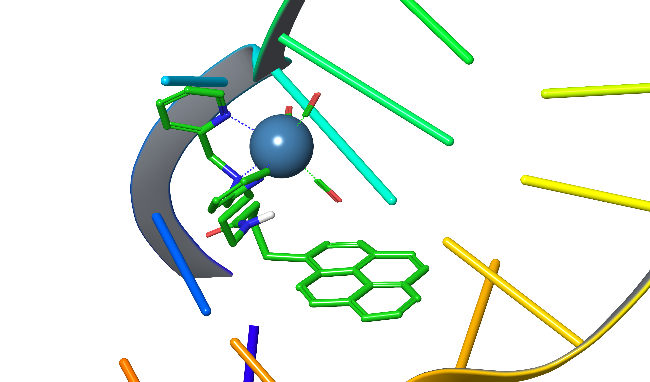

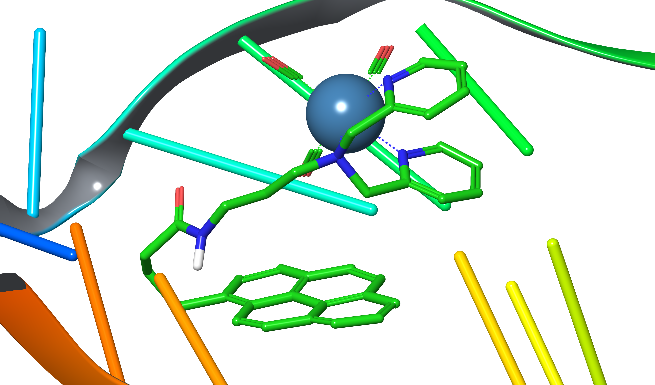
6 Å 9 Å 14 Å

**Fig S3.** **RDF of Tc with respect to DNA from MD simulations of the D models.**


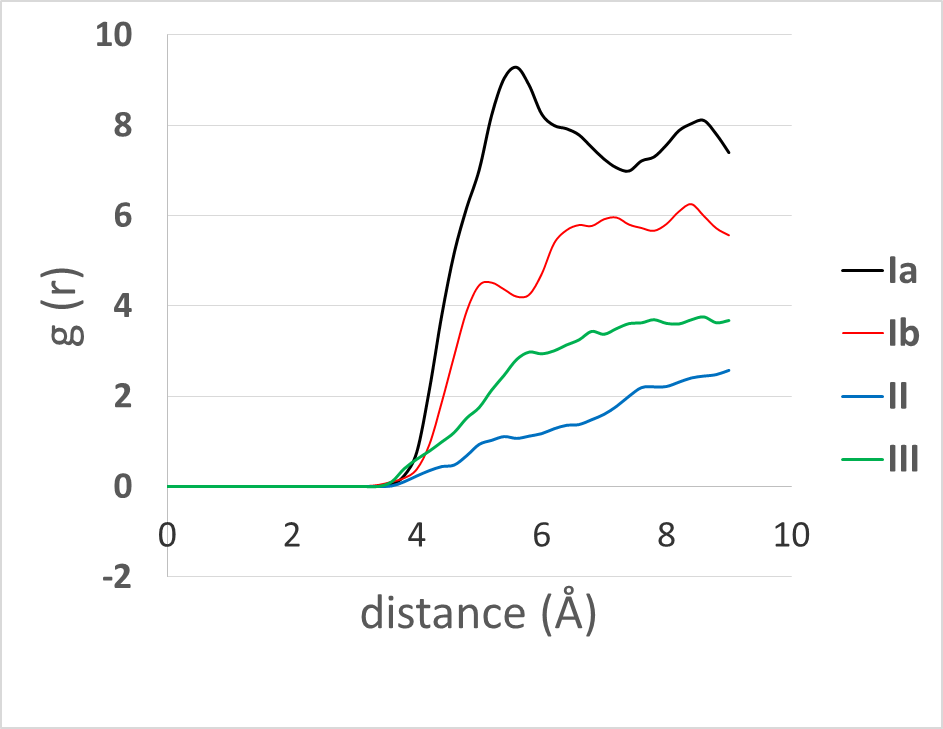


Higher intensity of g(r) at a distance value (for instance 5 Å) indicates more proximity to DNA. According to this, the order of more proximity of Tc to DNA is:

Compound **Ia** > Compound **Ib** > Compound **III** > Compound **II**


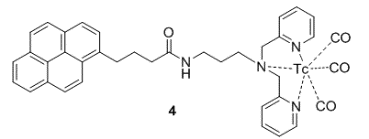

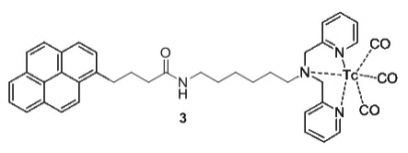

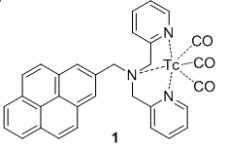

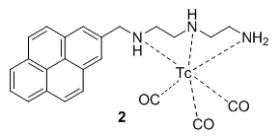


**References**

1. Hay PJ, Wadt WR. Ab initio effective core potentials for molecular calculations. Potentials for K to Au including the outermost core orbitals. J Chem Phys. 1985;82: 299–310.

2. Bochevarov AD, Harder E, Hughes TF, Greenwood JR, Braden DA, Philipp DM, et al. Jaguar: A high‐performance quantum chemistry software program with strengths in life and materials sciences. Int J Quantum Chem. 2013;113: 2110–2142.

3. Berendsen HJC, Postma JPM, van Gunsteren WF, DiNola A, Haak JR. Molecular dynamics with coupling to an external bath. J Chem Phys. 1984;81: 3684. doi:10.1063/1.448118

4. Zhang N, Lin C, Huang X, Kolbanovskiy A, Hingerty BE, Amin S, et al. Methylation of cytosine at C5 in a CpG sequence context causes a conformational switch of a benzo [a] pyrene diol epoxide-N 2-guanine adduct in DNA from a minor groove alignment to intercalation with base displacement. J Mol Biol. 2005;346: 951–965.

5. Bowers KJ, Chow E, Xu H, Dror RO, Eastwood MP, Gregersen BA, et al. Scalable algorithms for molecular dynamics simulations on commodity clusters. IEEE; 2006. pp. 43–43.

6. Guo Z, Mohanty U, Noehre J, Sawyer TK, Sherman W, Krilov G. Probing the α‐Helical Structural Stability of Stapled p53 Peptides: Molecular Dynamics Simulations and Analysis. Chem Biol Drug Des. 2010;75: 348–359.

7. Shivakumar D, Williams J, Wu Y, Damm W, Shelley J, Sherman W. Prediction of absolute solvation free energies using molecular dynamics free energy perturbation and the OPLS force field. J Chem Theory Comput. 2010;6: 1509–1519.
